# MeCP2 recognizes cytosine methylated tri-nucleotide and di-nucleotide sequences to tune transcription in the mammalian brain

**DOI:** 10.1371/journal.pgen.1006793

**Published:** 2017-05-12

**Authors:** Sabine Lagger, John C. Connelly, Gabriele Schweikert, Shaun Webb, Jim Selfridge, Bernard H. Ramsahoye, Miao Yu, Chuan He, Guido Sanguinetti, Lawrence C. Sowers, Malcolm D. Walkinshaw, Adrian Bird

**Affiliations:** 1 Wellcome Trust Centre for Cell Biology, University of Edinburgh, Edinburgh, United Kingdom; 2 School of Informatics, University of Edinburgh, Edinburgh, United Kingdom; 3 Institute of Genetics and Molecular Medicine, University of Edinburgh, Edinburgh, United Kingdom; 4 Department of Chemistry and Institute for Biophysical Dynamics, University of Chicago, Chicago, Illinois, United States of America; 5 Howard Hughes Medical Institute, University of Chicago, Chicago, Illinois, United States of America; 6 Department of Pharmacology and Toxicology, University of Texas Medical Branch, Galveston, Texas, United States of America; 7 Centre for Translational and Chemical Biology, University of Edinburgh, Edinburgh, United Kingdom; Albert Einstein College of Medicine, UNITED STATES

## Abstract

Mutations in the gene encoding the methyl-CG binding protein MeCP2 cause several neurological disorders including Rett syndrome. The di-nucleotide methyl-CG (mCG) is the classical MeCP2 DNA recognition sequence, but additional methylated sequence targets have been reported. Here we show by *in vitro* and *in vivo* analyses that MeCP2 binding to non-CG methylated sites in brain is largely confined to the tri-nucleotide sequence mCAC. MeCP2 binding to chromosomal DNA in mouse brain is proportional to mCAC + mCG density and unexpectedly defines large genomic domains within which transcription is sensitive to MeCP2 occupancy. Our results suggest that MeCP2 integrates patterns of mCAC and mCG in the brain to restrain transcription of genes critical for neuronal function.

## Author summary

Rett Syndrome is a severe neurological disorder found in approximately 1:10.000 female births. The gene causing most cases of Rett Syndrome has been identified as methyl-CG binding protein 2 (MeCP2) which is an epigenetic reader protein, classically characterized as binding to CpG methylated (mCG) di-nucleotides. Although much research has focused on the binding capacities of MeCP2, its exact mode of action is still controversial. Here we show, that in addition to the classical mCG motif, frequently occurring mCAC tri-nucleotides are also bound by MeCP2. We additionally discover large genomic regions of high mCG + mCAC density that contain neuro-disease relevant genes sensitive to MeCP2 loss or overexpression. Our results re-emphasize MeCP2’s original proposed function as a transcriptional repressor whose purpose is to maintain the delicate balance of neuronal gene expression.

## Introduction

Methylation at the C5 position of cytosine is an epigenetic mark implicated in gene regulation and disease [1]. In mammals, DNA methylation occurs most often in a CG di-nucleotide context, but in neuronal cells and embryonic stem cells (ESCs) mCA is detected at significant levels [2,3]. Like mCG, mCA is negatively correlated with transcript abundance, hinting at a repressive function in the brain [2,3]. Highest levels of non-CG methylation are observed in the human and mouse brain, where mCA accumulates postnatally at the same time as a phase of active synaptogenesis [3]. In mice, the increase in neuronal mCA coincides with accumulation of the DNA methyltransferase Dnmt3a [3], which is able to methylate CA, albeit at a low rate [4–7]. Brain mCA occurs most frequently in the tri-nucleotide mCAC [2,8], whereas in ESCs mCAG is the preferred sequence context. The biological significance of these preferences has yet to be elucidated [2,3].

A potential mechanism for interpreting the DNA methylation signal is the recruitment of methyl-CG binding domain (MBD) proteins including MeCP2, MBD1, MBD2 and MBD4 [9]. Of these, MeCP2 has attracted most attention as mutations involving the *MECP2* gene cause the X-linked autism spectrum disorder Rett syndrome [10] and *MECP2* duplication syndrome [11]. Rett missense mutations cluster in two domains of MeCP2: the MBD and the NCoR/SMRT co-repressor Interaction Domain (NID) [12,13]. These observations raise the possibility that loss of binding to methylated DNA and/or failure to recruit the NCoR/SMRT repressor complex are primary causes of Rett syndrome. MeCP2 has a high affinity *in vivo* and *in vitro* for binding to mCG [14–16], but the determinants of its targeting to DNA have recently diversified to include mCA, whose postnatal accumulation is paralleled by an increase in MeCP2 protein [2,3]. In addition, it has been reported that MeCP2 binds to hydroxymethylcytosine (hmC), the major oxidized form of mC, which is abundant in neurons [17]. Finally, there have been suggestions that MeCP2 can bind chromatin in a DNA methylation-independent manner [15,18–20].

The mutational spectrum and biochemical interactions of MeCP2 suggest that it behaves as a transcription repressor [13,21]. Changes in the mouse brain transcriptome when the protein is absent, however, involve both up- and down-regulation of genes [17,22,23]. Accordingly MeCP2 has been proposed to act as an activator of transcription or as a multifunctional hub that effects diverse aspects of cellular metabolism [12,24,25]. An additional model proposes that MeCP2 primarily functions by globally modifying the architecture of chromatin via multifaceted interactions with DNA [16,19]. An inverse correlation between levels of CA methylation and expression of long genes has recently re-emphasized the role of MeCP2 in transcriptional inhibition [26]. On the other hand a separate study reported that mCA is enriched within genes that are mis-regulated regardless of the direction of the transcriptional change in response to MeCP2 depletion or excess [27]. Despite progress, therefore, a consensus view regarding the role of MeCP2 in transcriptional regulation has been elusive.

Here we define the DNA binding specificity of MeCP2 using *in vitro* and *in vivo* approaches. We show for the first time that MeCP2 binding to non-CG methylated sites is primarily restricted to the tri-nucleotides mCAC or hmCAC *in vitro* and *in vivo*. Modeling based on the X-ray structure of the MBD of MeCP2 suggests that mCAC and mCG interact with a common protein conformation and may therefore lead to indistinguishable down-stream biological effects. MeCP2 binding across the adult brain genome reveals long genomic domains of high and low occupancy that match the distribution of mCAC + mCG binding sites. The results have important implications for regulation of gene expression, as we uncover a strong correlation between MeCP2 binding, mCAC + mCG density and the direction of gene mis-regulation when MeCP2 is absent or over-expressed. Our findings shed new light on the binding properties of MeCP2 and implicate MeCP2 as a global negative modulator of neuronal transcription.

## Results

### MeCP2 recognizes modified di- and tri-nucleotide sequences

**Fig 1:**
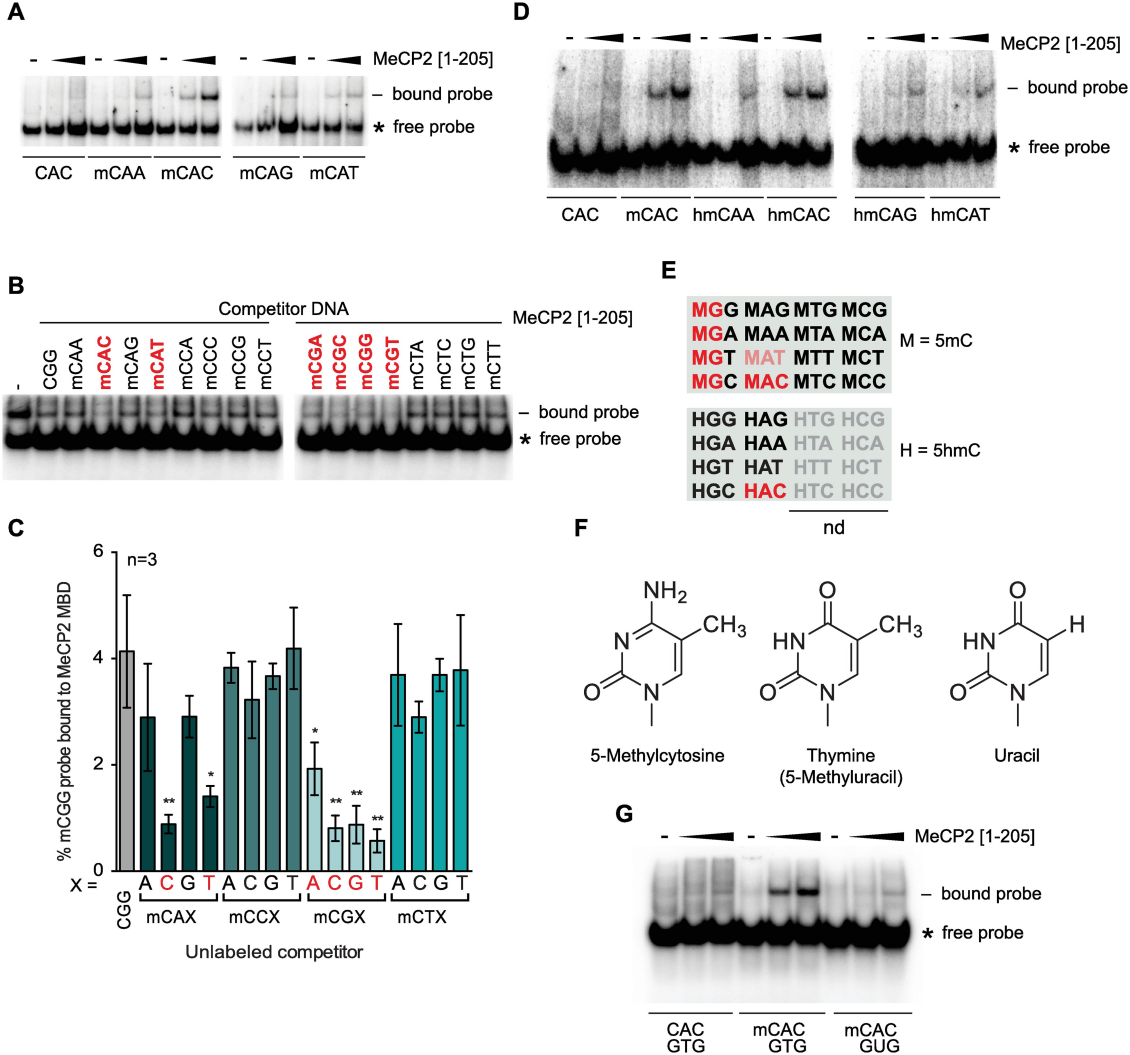
MeCP2 binds mCAC and hmCAC *in vitro*. (A) EMSA using no protein (-) or varying amounts of MeCP2 [1–205] with a probe (S1 Table) containing a centrally methylated C in a CAX context. Gap indicates separate gels. (B) EMSA using MeCP2 [1–205] or no added protein (-) in the presence of excess unlabeled unmethylated competitor DNA (CGG) or methylated competitor DNA (mCXX). Labeled probe contains mCGG. Red denotes strongest competition. (C) Quantification of (B). Three individual experiments were averaged. Red denotes most significant competition. Significance was calculated in relation to unmethylated CGG (grey bar). Error bars represent ± SD. Students unpaired t-test: * p<0.05; ** p<0.01. (D) EMSA using no protein (-) or varying amounts of MeCP2 [1–205] with a probe containing a centrally hydroxymethylated C in a CAX triplet context. (E) Summary of MeCP2 binding motifs *in vitro*. M = 5-methylcytosine, H = 5-hydroxymethylcytosine. Bright red: strong binding; pale red: weaker binding; grey: not tested. (F) Structure of 5-methylcytosine, thymine and uracil. Note that thymine and uracil are distinguishable by a methyl group on position 5 of the pyrimidine ring. (G) EMSA using no (-) or varying amounts of MeCP2 [1–205] to assess the influence of the methyl group of thymine on binding. *: free probe;—: bound probe.

The predominant methylated sequence is the di-nucleotide CG, but in adult brain mCA [3] and hmCG [17,28] are implicated as binding partners of MeCP2. A recent study concluded that in addition to mCG, MeCP2 binds both mCA and hmCA [26] and confirmed earlier reports that hmCG is a low-affinity binding site for MeCP2 [26,29–31]. In addition MeCP2 has been reported to bind *in vitro* to DNA in which every cytosine was substituted with hmC [17]. To comprehensively analyze the DNA sequence determinants of MeCP2 binding *in vitro* we performed EMSA analysis. As full-length MeCP2 binds to DNA poorly *in vitro*, most likely due to masking of the MBD by unstructured C-terminal sequences, these experiments utilized the 1–205 N-terminal domain of MeCP2 which contains the MBD and only robustly detectable AT-hook [32]. The MBD is the dominant DNA binding motif in MeCP2 and most missense mutations that cause RTT disrupt its ability to bind methylated DNA. Unexpectedly, the EMSA data revealed a novel constraint on MeCP2 binding, as the third base following mCA strongly affected MeCP2 binding affinity *in vitro* (Fig 1A). Probes containing the mCAC tri-nucleotide sequence bound with high affinity to MeCP2, whereas probes containing mCAA, mCAG and mCAT bound much less strongly. This result was confirmed in EMSA experiments using all possible mCXX tri-nucleotide sequences as unlabeled competitors against a labeled mCGG-containing probe (Fig 1B). Quantification showed that mCAC and, to a lesser extent mCAT, are both effective competitors, but mCAG and mCAA compete no better than non-methylated control DNA (Fig 1C). All mCGX oligonucleotide duplexes competed strongly indicating that the base following mCG on the 3’ side does not have a large effect on binding, although we note that mCGA was reproducibly a weaker competitor than mCGC, mCGG or mCGT. Neither mCCX nor mCTX tri-nucleotides had a significant affinity for MeCP2 *in vitro*.

As hmCA is reported to bind MeCP2 *in vitro* [26], we asked whether the third base is also important for hmC binding. Using hmCXX tri-nucleotides as probes in EMSAs, we found that hmCAC bound with a much higher affinity than hmCAA, hmCAG and hmCAT DNA (Fig 1D). It is notable that the great majority of hmC in the brain and elsewhere is in the hmCG di-nucleotide, with hmCAC being extremely rare [3]. The DNA binding specificity of MeCP2-MBD deduced from these *in vitro* experiments is summarized in a matrix of di- and tri-nucleotide sequences that bind to MeCP2 (red lettering, Fig 1E).

A notable feature of the mCAC binding site is that mC is only present on one strand, whereas mCG possesses symmetrically placed mC moieties. Within mCAC the position normally taken by mC across the dyad is occupied by thymine, which is effectively 5-methyluracil (Fig 1F). We therefore speculated that the mC methyl group in the MeCP2-mCAC complex is provided by thymine. To test this, we replaced thymine with uracil in the labeled probe and performed EMSA analysis (Fig 1G). Loss of the thymine methyl group abolished binding to MeCP2. The data suggest that a symmetrical pair of 5-methyl pyrimidines, one of which is mC, offset by one base pair is an essential pre-requisite for MBD binding to DNA. This finding also raises the possibility that the mode of binding to mCAC and mCG may be similar (see Discussion section for informal structural modeling based on this possibility).

### MeCP2 binding specificity *in vivo*

**Fig 2:**
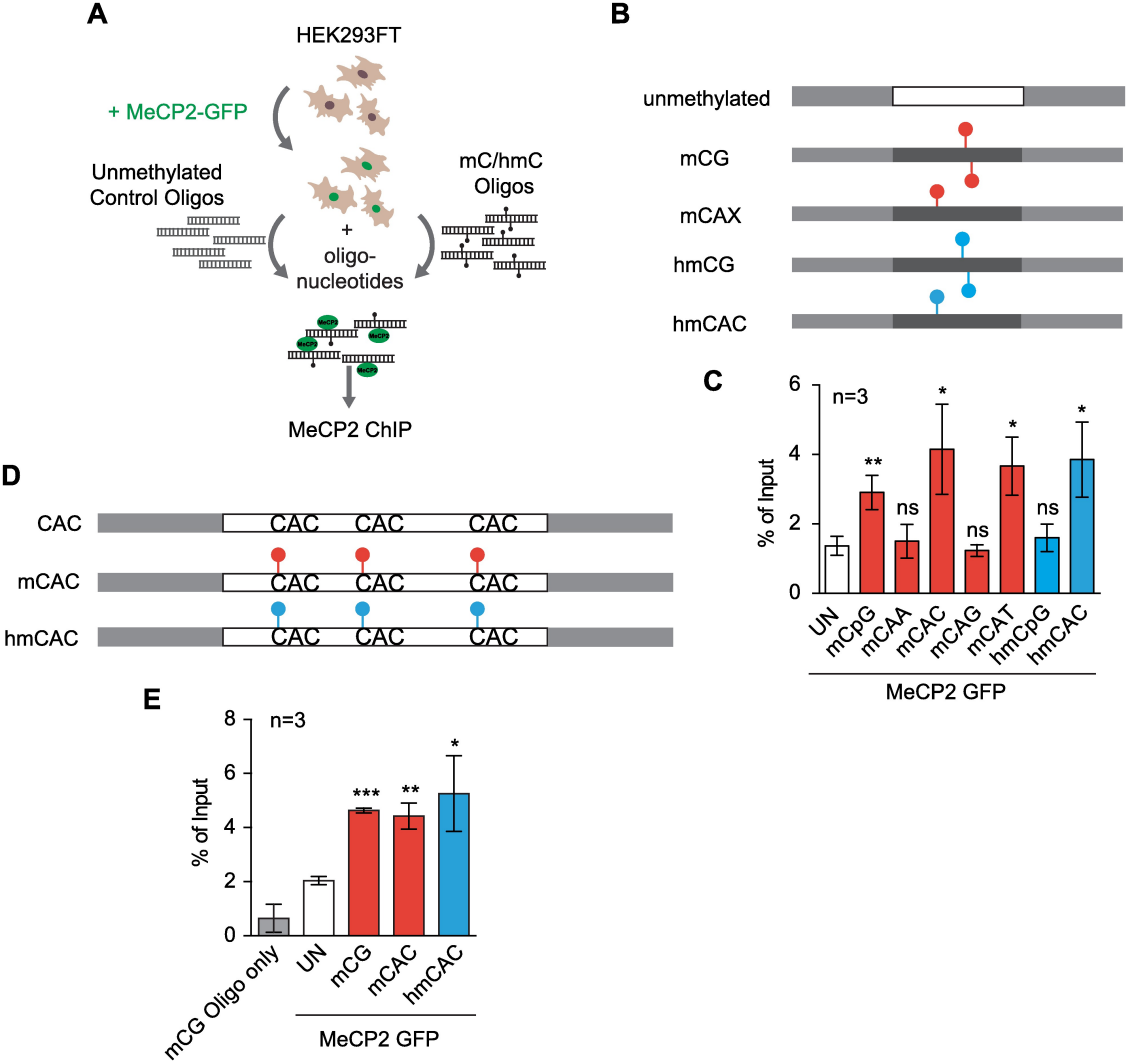
Full-length MeCP2 binds mCAC and hmCAC *in vivo*. (A) Schematic of *in vivo* transfection assay in HEK293FT cells. (B) Differentially modified oligonucleotide derived from the mouse *Bdnf* locus. Light grey: T3 and M13-20 adapters; red circles: mC; blue circles: hmC. See also S1 and S2 Tables. (C) Real Time PCR of *in vivo* transfection assay in triplicate where WT MeCP2-GFP was co-transfected with oligonucleotide as described in (B). (D) Differentially modified oligonucleotide at three CAC sites. See also S1 and S2 Tables. Light grey: T3 and M13-20 adapters; red circles: mC; blue circles: hmC. (E) Real Time PCR of *in vivo* transfection assay in triplicates where WT MeCP2-GFP was co-transfected with oligonucleotides as described in (D). Real Time PCR results are presented as % of Input (red bars: mC; blue bars: hmC, white bars: unmethylated; grey bar: mCG oligonucleotide transfected without prior transfection of MeCP2-GFP as a background control). Error bars represent ± SD. Significance was calculated in relation to unmethylated oligonucleotide transfections (white bars). Students unpaired t-test: ns p>0.05; * p<0.05; ** p<0.01; *** p<0.001.

To determine whether the binding specificities established *in vitro* apply to full-length MeCP2 protein in living cells, we developed a novel assay using transfection followed by chromatin IP (ChIP) [33]. Synthetic DNA duplexes containing specific cytosine modifications were transfected into HEK293 cells expressing human full length MeCP2 tagged with GFP (Fig 2A and S1A and S1B Fig). Levels of endogenous MeCP2 in these cells are negligible and therefore do not interfere with the assay (S1C Fig). We tested oligonucleotide duplexes containing a single modified cytosine in either a mCG, hmCG, mCAX or hmCAX context (Fig 2B). The results showed that mCAC, mCAT, hmCAC and mCG all bound MeCP2 efficiently, whereas hmCG, mCAA and mCAG binding was indistinguishable from background binding to non-methylated DNA (Fig 2C). The same outcome was seen with different DNA sequences containing three CAC motifs per oligonucleotide, either all unmodified, all methylated or all hydroxymethylated (Fig 2D and 2E). These *in vivo* results with full-length protein reinforce the evidence obtained using EMSAs that MeCP2 requires specific tri-nucleotide settings to recognize mC or hmC in a non-CG context. While *in vivo* and *in vitro* data match in nearly all respects, we noted a quantitative difference regarding mCAT binding, which bound relatively weak in EMSAs, but was robustly detected in the transfection assay (Fig 2C).

### DNA sequence specificity of MeCP2 binding in adult mouse brain

**Fig 3:**
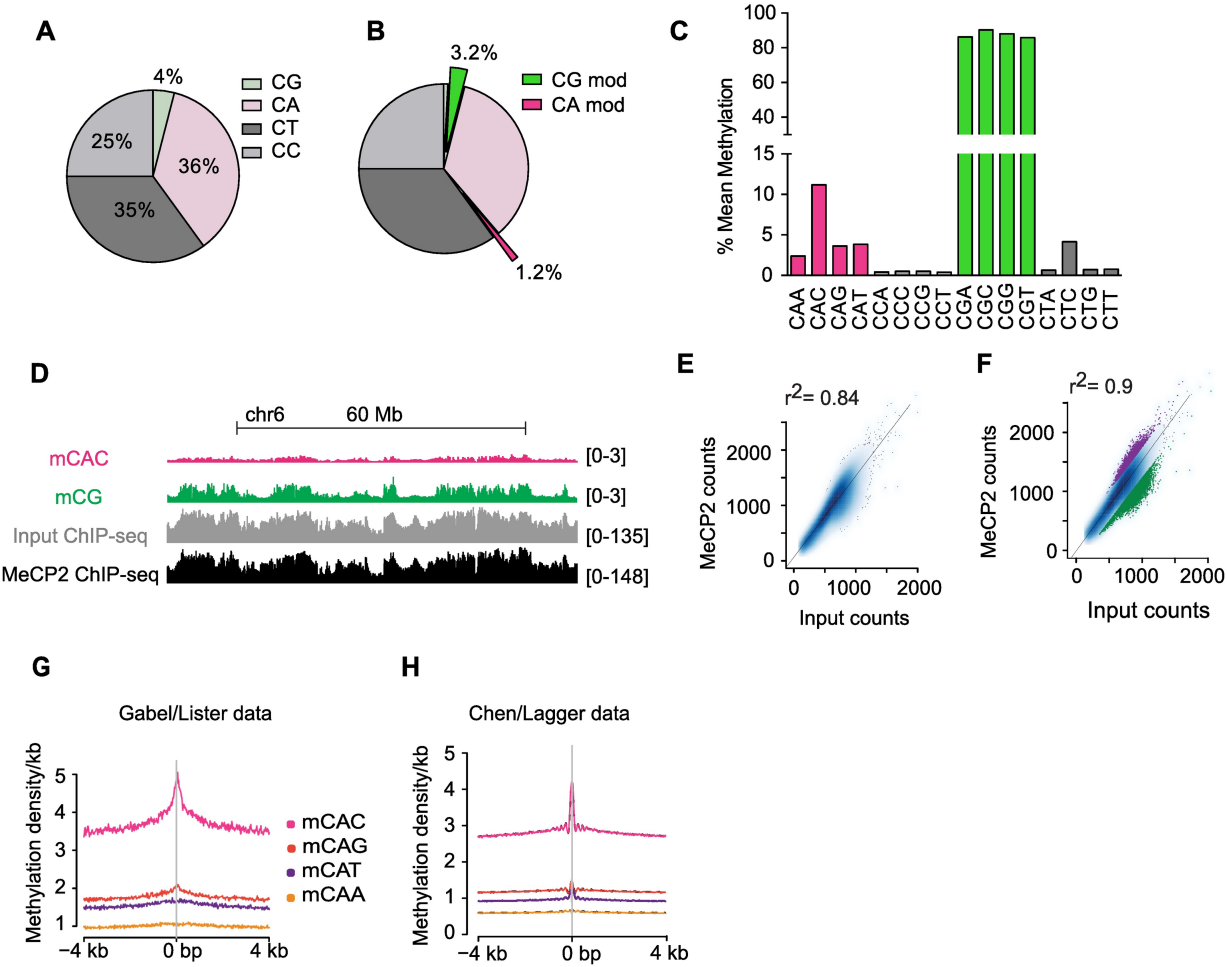
DNA methylation and MeCP2 binding in the mouse brain. (A) Pie chart showing CX frequencies in the mouse genome (dataset WGBS of sorted neurons from this study). (B) Pie chart showing modified (mC and hmC) CX frequencies in brain neuronal nuclei as determined by WGBS (dataset WGBS of sorted neurons from this study). (C) Mean methylation levels (%) of CXX in brain neuronal nuclei based on WGBS of sorted neurons (dataset from this study). (D) IGV browser screenshot of mCAC, mCG, MeCP2 ChIP-seq and corresponding Input DNA based on sequence reads. Data represent a 100 Mb region of chromosome 6 (datasets from [3,27]). (E) Correlation between sequence coverage of MeCP2 ChIP-seq and corresponding Input DNA in 10 kb windows. Fitting a linear model (MeCP2 ~ Input) yields a coefficient of determination of 0.84 (dataset from [27]). (F) As for (E) but highlighting relative depletion (4.1% of windows; green) and relative enrichment (1.7% of windows; purple). When these outliers are excluded, 90% of the variability of the MeCP2 signal in the remaining binned windows is explained by sequence bias (Input coverage) (dataset from [27]). (G-H) Enrichment of mCAC at summits of MeCP2 binding using ChIP-seq and WGBS datasets from matching brain regions ([3,26] (G) and [27] and this study (H)).

To test whether the DNA binding specificities established *in vitro* and in transfected cells also apply in native tissues, we analyzed MeCP2 ChIP-seq and whole genome bisulfite (WGBS) datasets derived from adult mouse brain (references [3,26,27] and WGBS from sorted neurons and hypothalamus [this study]). CG is under-represented in the mouse genome (~4% of CX), but highly methylated (~80%), whereas CA is the most abundant CX di-nucleotide (36% of CX), but even in brain only a small fraction of CA is methylated (<2%) (Fig 3A–3C). Bisulfite analysis of neurons sorted by NeuN staining as described in reference [16] confirmed previous reports that CAC is the most methylated tri-nucleotide [2,8], being ~12% methylated (Fig 3C). The finding that the MeCP2 tri-nucleotide binding specificity matches the most abundantly methylated non-CG sequence in brain encourages the view that this interaction is biologically relevant.

Previous ChIP-seq analyses have concluded that MeCP2 read coverage tracks the density of CG methylation [15,16,26,27]. Re-analysis of several MeCP2-ChIP data sets for which the antibody used has been rigorously verified, indicates, that the profile of Input–that is, DNA derived from the fragmented chromatin sample used for ChIP–is closely similar to that of MeCP2 (Fig 3D). Using a ChIP-seq dataset for hypothalamus in which replicate Input and ChIP samples are sequenced at high depth [27], we fitted a linear model to predict MeCP2 read coverage from Input reads alone and found a coefficient of determination of 0.84 (Fig 3E). If we removed windows with relatively increased (purple) or decreased (green) MeCP2 read coverage (Fig 3F) and analysed only the remaining 94% of the genome, the variance in MeCP2 signal was 90% predictable by the Input coverage. This means that MeCP2 is relatively uniformly distributed across most of the genome at this resolution. These results are in line with the high binding site frequency and a previous report that the number of MeCP2 molecules in mature neurons is sufficient to almost ‘‘saturate” mCG sites in the genome [16]. Given the similarity between ChIP and Input, we used Input corrected MeCP2 signals (log2(MeCP2 ChIP/Input)) and complemented genome-wide analysis by examining regions that deviate from the Input profile regarding enrichment or depletion of MeCP2. First, we investigated genomic regions that are depleted of potential binding sites, e.g. unmethylated CpG islands (CGIs). As the ChIP dataset was derived from mouse hypothalamus, we derived a DNA methylome for this brain region by performing WGBS on three biological replicates (see Online Methods). Using ChIP and DNA methylation datasets from the same brain regions, we observed a pronounced drop in the log2(MeCP2 ChIP/Input) signal across CGIs in both datasets, in line with previous analyses [16,26,27] (S2A Fig). We next examined regions where the MeCP2 signal was higher than expected by applying the MACS [34] tool to detect summits of MeCP2 binding peaks relative to Input [26]. As expected, the di-nucleotides mCG and mCA showed a sharp peak at MeCP2 ChIP summits in the hypothalamus dataset (S2B Fig and [26]). Further, the tri-nucleotide mCAC, but not other mCAX tri-nucleotides, coincided strikingly with MeCP2 peak summits, confirming that mCAC provides a focus for MeCP2 binding (Fig 3G and 3H). Using random regions as a negative control, we did not detect any sequence or methylation dependency (S2C Fig). Regarding targeting of mCAT, which bound relatively weakly in EMSA, but strongly in the transfection assay, the ChIP-seq data suggest that this is a low affinity binding site in native brain (Fig 3G and 3H).

**Fig 4:**
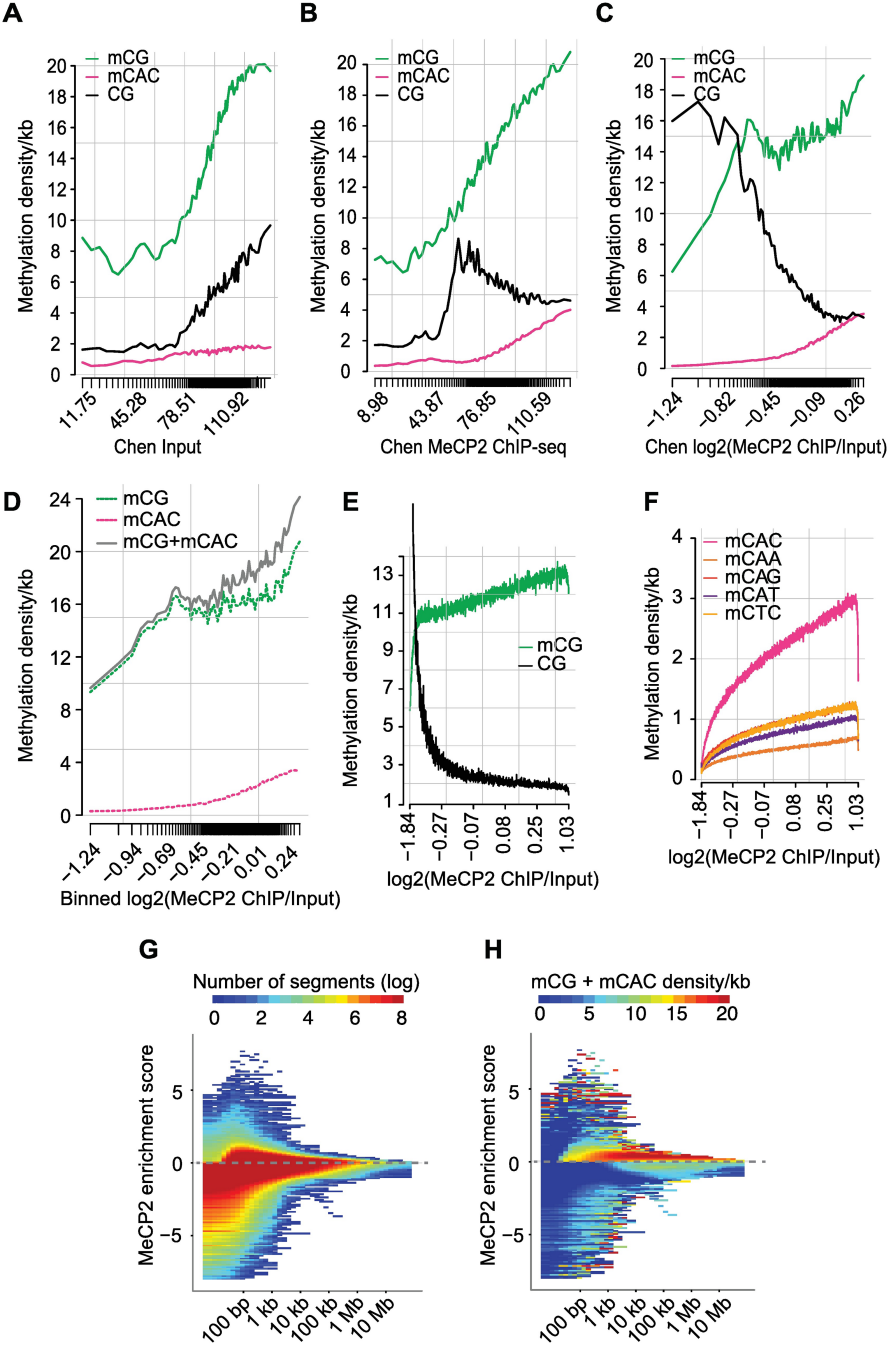
MeCP2 binds to mCG and mCAC in large domains extending beyond the gene scale. (A) Density of mCG/mCAC or unmethylated CG per 1kb as a function of ChIP-seq Input coverage in protein-coding genes. The density of tick marks on the x-axis represents the distribution of genes with respect to Input coverage (datasets from [27] and hypothalamus WGBS from this study). (B) As in (A) but for MeCP2 ChIP-seq read coverage on the x-axis. (C) As in (A and B) but corrected for Input and shown as log2(MeCP2 ChIP/Input) (datasets from [27] and hypothalamus WGBS from this study). (D) As in (C) showing additionally the combined density of mCG + mCAC sites (grey line) (datasets from [27] and hypothalamus WGBS from this study). (E) Relationship between mCG and unmethylated CG density/kb and MeCP2 occupancy corrected for Input in genome-wide 1 kb windows (datasets from [27] and hypothalamus WGBS from this study). (F) Relationship between mCAX and, as a negative control, mCTC density/kb and MeCP2 occupancy corrected for Input in genome-wide 1 kb windows (datasets from [27] and hypothalamus WGBS from this study). (G-H) Domains of MeCP2 enrichment and depletion as determined by MSR (datasets from [27] and hypothalamus WGBS from this study). Grey dotted line indicates zero. (G) Heatmap showing number of segments binned by their scale (x-axis) and MeCP2 enrichment scores (y-axis). X-axis shows the median lengths of segments found with a given scale. Positive scores on the y-axis indicate MeCP2 enrichment; negative scores indicate MeCP2 depletion. The plot is colored according to number of segments. (H) Heatmap showing number of segments binned by scale and scored as in (G). The plot is colored according to combined mCG + mCAC density.

To further explore MeCP2 occupancy *in vivo*, we analyzed MeCP2 binding preferences in protein coding genes. In the Input sample, both mCG density and the density of unmethylated CGs strongly correlated with coverage revealing a methylation-independent CG sequencing bias in both hypothalamus and cortex datasets (Fig 4A and S3A Fig). However, MeCP2 ChIP coverage was clearly sensitive to DNA methylation, with mCG being positively correlated, while unmethylated CG density was anti-correlated (Fig 4B and S3B Fig). Importantly, DNA methylation-sensitivity was also observed in the Input-corrected signal (log2(MeCP2 ChIP/Input)), strongly supporting the view that mCG and mCAC are targets for MeCP2 binding (Fig 4C and S3C Fig). To complement the analysis of protein coding genes, we found once again that the Input-corrected signal is strongly correlated with the density of both mCG and mCAC (green and pink lines) but gives the best correlation upon summation of both individual binding motifs (grey line) (Fig 4D). We identified similar binding preferences when adopting a sliding window approach to 1kb regions across the genome (Fig 4E and 4F). This mCG binding preference was independent of the third DNA base (S3D Fig). In agreement with the *in vitro* and *in vivo* results reported above, the density of mCAC also correlated strongly with increasing MeCP2 enrichment, whereas a much weaker trend was observed for other methylated tri-nucleotide sequences (Fig 4F and S3E and S3F Fig). In contrast to published reports [17,35], we found no evidence for MeCP2 binding to hmCG in native brain (S3G and S3H Fig). We also failed to detect MeCP2 binding to unmethylated C’s in any sequence context (S3I–S3L Fig). Taken together, our analysis of ChIP data strongly supports the view that MeCP2 binding is determined by the combined density of mCG and mCAC sites.

Given the high abundance and global distribution of MeCP2 in the neuronal genome, we looked for domains of MeCP2 occupancy that might reflect long-range variation in binding site abundance. To avoid pooling data in arbitrary windows, we used a multiscale representation method (MSR) that identifies patterns of signal enrichment or depletion across scales spanning several orders of magnitude [36]. MSR identified a large number of long domains moderately enriched for MeCP2 of up to 1 Mb in length, indicating that regions of high MeCP2 occupancy extend beyond the scale of a single gene (Fig 4G). These regions share common sequence features, in particular high mCG and mCAC densities (Fig 4H and S3M and S3N Fig). We also identified many short regions that have high GC content (<1 kb) but are strongly depleted in MeCP2 binding. As expected, these latter regions significantly overlap with CGIs (S3O Fig). Lastly, we found a group of relatively long regions (10 kb—1 Mb) which are moderately depleted in MeCP2 binding (MeCP2 enrichment score < 0 to -2). These regions are relatively enriched for mCG but lack mCAC (Fig 4H and S3M–S3O Fig).

As part of this binding site analysis we re-visited an earlier report that used SELEX to demonstrate MeCP2 binds preferentially to mCG flanked by an AT-rich run of 4–6 base pairs *in vitro* [37]. To look for this preference in brain, we asked whether isolated mCG and mCAC flanked by a run of 4 or more A or T base pairs within 13 base pairs showed greater MeCP2 ChIP-seq signal than sequences lacking an AT-run. In summary, we find no evidence for an effect of AT-flanks on MeCP2 binding site occupancy (S3P and S3Q Fig). We speculate that SELEX, which requires repeated cycles of MBD binding followed by PCR amplification, has detected a real but weak preference that is of questionable biological relevance.

### The relationship between MeCP2 occupancy and gene expression

The robust association of MeCP2 with both methylated sites in the genome and the co-repressor complex NCoR [13] suggests that the protein can function to inhibit transcription. If so, a relationship would be expected between MeCP2 occupancy and the transcriptional mis-regulation when MeCP2 is either depleted by deletion of the gene (KO) [38] or over-expressed (OE) [39,40]. Before making use of published datasets for mouse hypothalamus [27], we first asked using HPLC whether the absence or overexpression of MeCP2 alters total RNA levels. Previous studies using MeCP2-deficient neurons differentiated *in vitro* from mouse ES cells or human iPS cells reported reduced total RNA and transcriptional capacity [25,41], but comparable measurements in brains of MeCP2-deficient mice have not been reported. Using a sensitive RNA quantification technique, we observed that total RNA per cell in KO hypothalamus is reproducibly 15% lower than WT (S4A Fig). Overexpression of MeCP2, however, did not significantly affect total RNA. As whole cell RNA is ~98% ribosomal RNA, we asked whether mRNA levels were also reduced in KO hypothalamus. Quantitative RT-PCR (qPCR), using spiked-in *Drosophila* cells to control for experimental error and normalized to brain cell number in each sample (S4B Fig), confirmed that genes previously reported to be up- or down-regulated in MeCP2-deficient hypothalamus [27] were similarly mis-regulated in our samples (S4C–S4F Fig). We then measured the transcript abundance for three housekeeping genes and found that all were down-regulated by approximately 15% (S4G–S4I Fig). These results suggest that total RNA and mRNA levels are coordinately reduced and we have therefore applied this normalization to all hypothalamus RNAseq datasets. The mechanisms responsible for reducing total RNA, and whether this effect is a primary or secondary consequence of MeCP2 deficiency, are currently unknown.

**Fig 5:**
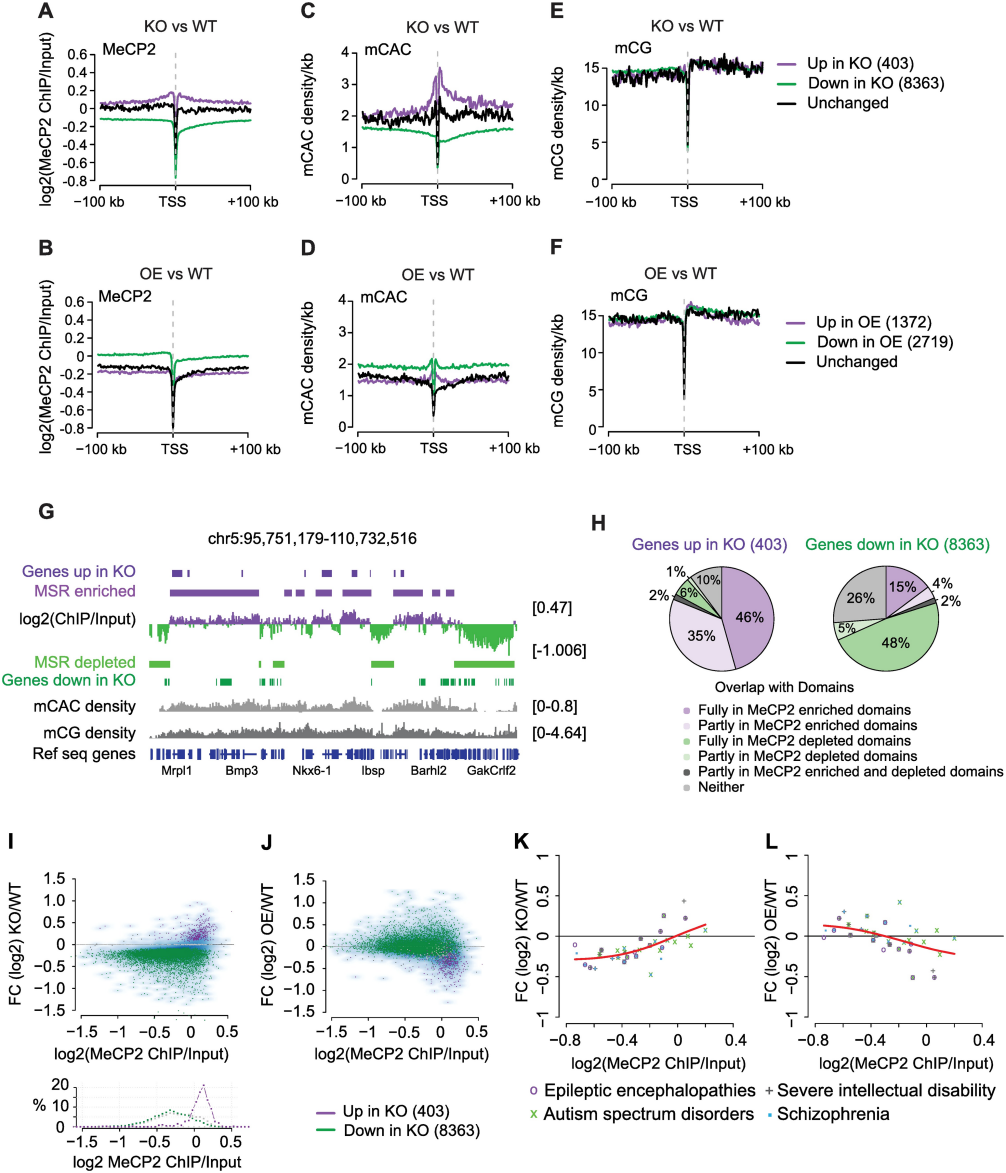
Genes show global down-regulation in KO, but MeCP2 enriched genes are up-regulated upon MeCP2 depletion and down-regulated when MeCP2 is overexpressed. Datasets [27] and hypothalamus WGBS from this study. (A-B) Aggregate WT MeCP2 occupancy plotted 100 kb up- and down-stream of the transcription start site (TSS) of genes that are up-regulated (purple), down-regulated (green) or unchanged (black) in (A) *Mecp2* KO or (B) *Mecp2* OE hypothalamus. (C-D) Methylation density per kb of CAC and (E-F) CG plotted 100 kb up- and down-stream of the TSS using the same gene sets as in (A) and (B) respectively. (G) IGV screenshot of chromosome 5 (chr5:95,751,179–110,732,516) showing unpruned MSR regions of scale 30, corresponding to a median segment length of 270 kb and 240 kb for MeCP2 enriched and MeCP2 depleted segments, respectively using datasets from [27] and hypothalamus WGBS from this study. (H) Pie charts showing percentage of up- (left) and down-regulated (right) genes and their overlap with MeCP2 enriched (purple) or depleted (green) domains using dataset [27] and hypothalamus WGBS from this study. (I-J) Transcriptional changes resulting from MeCP2 deficiency as a function of MeCP2 occupancy. Genes that are up-regulated in KO (purple) have high MeCP2 occupancy and show the strongest up-regulation in KO (I) and robust down-regulation in OE (J). FC = fold change. Histogram depicting the correlation between MeCP2 occupancy and % of genes that are up- (purple), down-regulated (green) and unchanged (grey). Dataset [27]. (K-L) MeCP2 occupancy and expression changes are well correlated at genes previously implicated in neurological diseases [42]: Spearman correlation coefficients of 0.67 for KO vs WT (K) and -0.5 for OE vs WT (L) Dataset [27].

We next examined gene expression by separating genes whose expression was increased, unaltered or decreased in response to changing levels of MeCP2. Normalizing ChIP signals against Input, we found that up-regulated genes in KO hypothalamus were within domains enriched in MeCP2, whereas down-regulated genes were within relatively MeCP2 depleted regions (Fig 5A). Unchanged genes showed an intermediate level of MeCP2 occupancy. The reciprocal result was seen in OE hypothalamus, as down-regulated genes had high MeCP2 occupancy, whereas up-regulated and unchanged genes bound relatively less MeCP2 (Fig 5B). This relationship, which was not observed in a previous analysis of this gene expression and ChIP-seq dataset, disappeared altogether if gene body binding of MeCP2 was normalized to binding levels in gene flanking regions [27]. Adjustment of the data in this way obscures the relationship between DNA methylation and gene expression because enhanced or depleted binding of MeCP2 is not confined to gene bodies, but extends up- and down-stream of the transcription start and end sites (Fig 5A and 5B). We also asked whether the increased MeCP2 occupancy measured by ChIP-seq in hypothalamus correlated with an elevated level of the two target sequences mCAC and mCG. The distribution of mCAC strikingly matched the pattern of MeCP2 binding (Fig 5C and 5D), but mCG, which occurs at much higher density, correlated less obviously (Fig 5E and 5F). Possible reasons for this difference are considered below (see Discussion).

The strong reciprocal relationship between MeCP2 occupancy and the direction of gene mis-regulation in KO and OE hypothalamus respectively, is compatible with the notion that MeCP2 binding is inhibitory to transcription. Excess MeCP2 preferentially inhibits genes with most binding sites whereas its depletion preferentially de-represses highly occupied genes. Elevated or depleted MeCP2 binding extended up- and down-stream of the TSS, suggesting that these genes are embedded within the extended MeCP2 domains identified by our MSR analysis (Fig 4G and 4H). This was confirmed by mapping MeCP2-enriched and depleted domains onto the genome in relation to mis-regulated genes (Fig 5G). Approximately 80% of genes up-regulated in KO hypothalamus were within or overlapped domains of high MeCP2 occupancy (dark and pale lilac), whereas only 19% of down-regulated genes were associated with MeCP2 enrichment (Fig 5G, 5H and S5A and S5B Fig).

In order to visualize the relationship between transcription and MeCP2 occupancy comprehensively, transcript fold change levels were plotted against MeCP2 ChIP signal normalized to Input (Fig 5I and 5J and S5C and S5D Fig). The 15% global reduction in mRNA and ribosomal RNA relative to DNA automatically means that in KO hypothalamus, expression of most genes (8,363) is reduced (Fig 5I, green). Despite this overall trend, however, a small number of genes (403) significantly increased their expression per cell compared with WT (Fig 5I, purple). These genes, which shared high MeCP2 occupancy (Fig 5I, lower panel), showed reciprocal behavior in OE hypothalamus where they were down-regulated (Fig 5J). This inverse relationship is readily apparent in a plot of gene expression changes in KO/OE hypothalamus (S5E Fig). De-regulated genes shared similar levels of mCG density, but only the up-regulated genes displayed increased mCAC (S5F and S5G Fig). We noted that the genes up-regulated in KO hypothalamus were significantly longer than average, in agreement with a report that long genes are preferentially up-regulated in MeCP2 KO mice [26] (S5H Fig) and most were implicated in brain function by gene ontology analysis (S5I Fig). In addition to this group of genes, a gene set that has been implicated in a variety of neurological disorders [42] also showed behavior compatible with repression by MeCP2, as the magnitude of mis-regulation in KO and OE hypothalamus correlated reciprocally with MeCP2 occupancy (Fig 5K and 5L).

## Discussion

### DNA methylation-dependent recognition sequences of MeCP2

In this study, we comprehensively analyzed the modified DNA sequences that determine MeCP2 binding. To ensure the reliability of our conclusions we used three independent experimental approaches: *in vitro* EMSA using amino acids 1–205 of MeCP2; *in vivo* ChIP in transfected cultured cells using full length MeCP2; and *in vivo* ChIP-seq of native MeCP2 in mouse brain. Three methylated DNA motifs consistently recruited MeCP2: mCG, mCAC and hmCAC. Interestingly, mCAC is the predominant methylated non-CG sequence in brain, comprising 15–30% of all methylated cytosine in sorted mouse neurons, probably due to the action of the *de novo* DNA methyltransferase Dnmt3a [2,4]. The tri-nucleotide mCAT gave inconsistent results in our assays; being well bound in the transfection assays, weakly bound *in vitro* and undetectably bound in brain. We speculate that overexpression in transiently transfected cells may have exaggerated an otherwise weak interaction. It is likely that the brain ChIP data give the most reliable indication of biologically relevant binding specificity. Although the hydroxymethylated sequence hmCAC binds MeCP2 in all assays, it is reportedly extremely rare in brain perhaps due to the preference of Tet enzymes for mCG as a substrate [3]. Given the inability of MeCP2 to bind hmCG and the rarity of hmCAC, it seems unlikely that hmC is a major target for MeCP2. The data in fact suggest a negative role, as oxidation of the abundant mCG methyl group by Tet enzymes would “switch off” MeCP2 binding, thereby preventing recruitment of corepressor complexes at this site.

Our data also indicate that the methylation required for MBD binding to DNA can be supplied on one strand by thymine rather than 5-methylcytosine. We previously observed that replacement of a mC at a methylated CG di-nucleotide with T, forming a T:G mispair, had a negligible effect upon the binding affinity of MeCP2 [29]. This indicates that hydrogen bonded base pairs are not essential and that the interaction is flexible enough to accommodate T:G wobble geometry. Here we report that in duplex DNA one pyrimidine-methyl group can be provided by either the mC or T, as replacement of T with U, which lacks the T methyl group, results in loss of MeCP2 binding.

**Fig 6:**
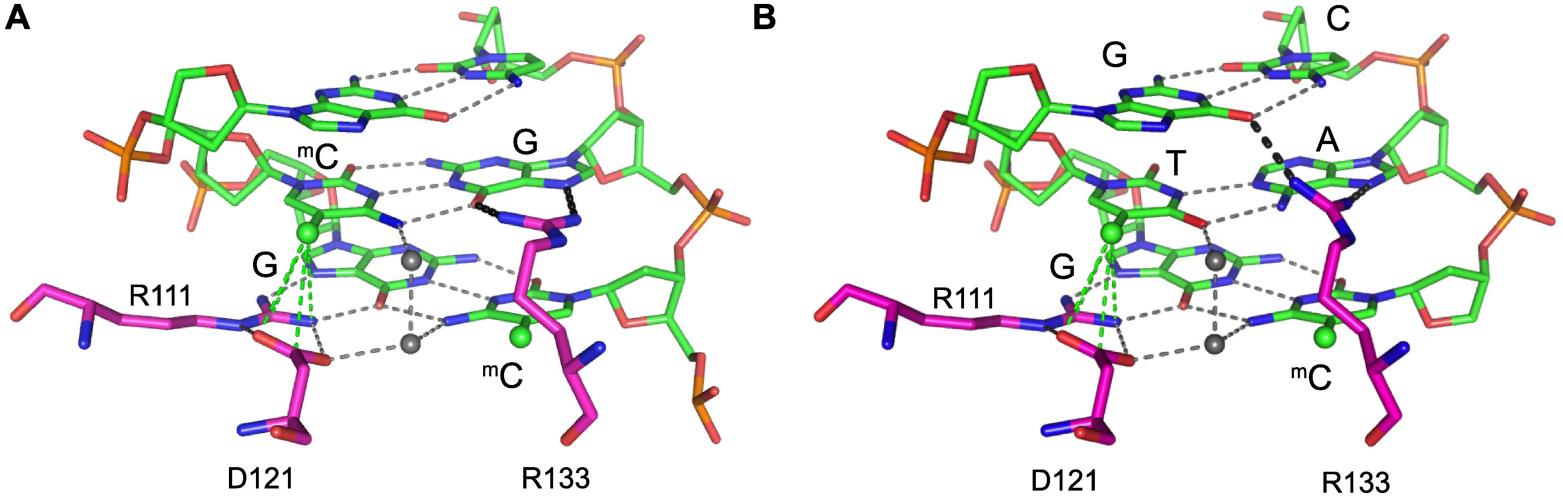
Hypothetical modeling of MeCP2 tri-nucleotide recognition suggests that flexibility may depend on the R133 side chain and require the methyl group of thymine. (A) X-ray structure of MeCP2 [77–167] interaction with the mCG di-nucleotide in double-strand DNA [43]. Critical amino acids R111, R133 and D121 are shown in pink. Green sphere: methyl group; grey sphere: water molecule; green dotted lines (from R111 and D121): favorable interactions; grey dotted lines: hydrogen bonds. (B) Modeling of the MeCP2 [77–167] interaction with double-strand DNA containing mCAC. Key as in (A) and black dotted lines: modeled interactions. See extended legend to S6 Fig for rationale.

To explore the structural basis for the tri-nucleotide specificity of MeCP2 binding, we asked whether the X-ray structure of the MeCP2-MBD [43] could suggest why mCAC or mCAT binding is permitted while mCAA, mCAG, mCCX and mCTX are forbidden. Surprisingly, informal modeling indicated that altering the conformation of only one amino acid side chain, R133, while leaving all other coordinates of the established X-ray protein structure unchanged, could hypothetically account for the observed interactions (Fig 6 and S6 Fig plus accompanying extended legend). Thus, the observed tri-nucleotide binding specificity of MeCP2 can hypothetically be explained with minimal perturbation of the established structure of the MBD-DNA complex. Although structure determination is essential to test these predictions, their potential impact on MeCP2 function is of interest. Formally, mCAC and mCG may be read by MeCP2 as independent signals with distinct biological outcomes. Alternatively, they may lead to functionally identical consequences when MeCP2 is bound. Favoring the second possibility, published evidence indicates that MeCP2 can repress transcription via mCH and mCG sites [2]. If, as our modeling implies, binding to either mCG or mCAC is accompanied by a minimal conformational shift in the MBD structure, we anticipate that the biological consequences of binding to either motif will be the same. Further experiments are required to test these conjectures rigorously.

### DNA methylation is the dominant determinant of MeCP2 binding in the brain

It was shown previously that DNA methylation is the primary determinant of MeCP2 binding in cultured cells by genetic ablation of the DNA methyltransferases [15,44]. DNA methylation-independent binding has been detected when the MBD is mutated [15], but as individuals lacking a functional MBD nevertheless exhibit severe RTT, the significance of this non-specific interaction is questionable. Our re-analysis of MeCP2 ChIP datasets greatly strengthens the argument that mC-directed binding is a critical function of MeCP2. Mapping bound MeCP2 *in vivo* has been problematic, as the high frequency of mCG and mCAC binding sites throughout the neuronal genome (one per ~100 bp) poses challenges for conventional ChIP-seq analysis. Unlike transcription factors, whose binding sites tend to be widely spaced compared with the size of chromatin-derived DNA fragments (200–500 bp), MeCP2 binding sites are short and frequent. As a result of this difference, only a minority of DNA fragments is immunoprecipitated by transcription factor antibodies, leading to recovery of discrete peaks, whereas in the case of MeCP2, most genomic DNA fragments contain mCAC and/or mCG (CGIs being a conspicuous exception), leading to a relatively uniform recovery of genomic DNA. Thus, the difference between Input and ChIP signal, which is the measure of MeCP2 density, provides an undulating continuum in which peaks are broad. In spite of these limitations, our careful normalization of ChIP versus Input reads detects a robust relationship between MeCP2 binding and the density of mCAC + mCG in brain nuclei.

Contrasting with this conclusion, a recent study using olfactory bulb neurons, reported that MeCP2 is enriched at non-methylated CGIs and that DNA methylation is a minor determinant of binding [20]. This conflicts with the findings of our study and several previously published MeCP2 ChIP experiments, all of which found a dramatic drop in MeCP2 binding at CGIs in cultured cells [15], whole mouse brain [16], hypothalamus [27], cortex and cerebellum [26] coupled with DNA methylation-dependent occupancy of the genome. It is notable that these reports of DNA methylation-dependence were achieved using a diversity of validated antibodies. One potential explanation for the discrepant recent results in olfactory bulb is that the rules governing MeCP2 binding in this region of the brain differ from those operating in the remainder of the nervous system. Alternatively, there may be a technical issue regarding antibody specificity or differential PCR amplification of ChIP and Input samples prior to sequencing that has led to inconsistent findings.

### Relationship between MeCP2 binding profiles and gene expression

A striking feature of the MeCP2 KO hypothalamus is the reduced level of total RNA, in agreement with reports in cultured mouse and human neurons [25,41]. The mechanism responsible is unknown, but one proposal is that MeCP2 is a direct global activator of transcription [24,25]. Arguing against this possibility, we found that most KO down-regulated genes lie in domains of low MeCP2 occupancy. Also, it might be expected that two-fold overexpression of an activator would lead to increased levels of RNA compared to WT, but this is not observed (S4A Fig). An alternative explanation is that reduced RNA reflects reduced cell size, perhaps as a secondary consequence of sub-optimal neuronal gene expression. In this case, the change in total RNA and the relative mis-regulation of genes within the transcriptome may be separate phenomena. Given the close relationship between gene mis-regulation, MeCP2 binding site density and MeCP2 occupancy, we favor the view that effects of MeCP2 concentration on the balance of neuronal gene expression are primary, whereas the downward shift in total RNA is a secondary effect. To test this rigorously it will be important to track down the origins of the total RNA deficiency.

By re-analyzing ChIP-seq and RNA-seq datasets from hypothalamus of WT mice, *Mecp2*-null mice and mice over-expressing MeCP2 we were unable to confirm reports that MeCP2 is more highly bound to the transcription units of mis-expressed genes regardless of up- or down-regulation [27]. Instead we found that MeCP2 is bound at higher levels within and surrounding genes that are up-regulated when MeCP2 is missing, or down-regulated by MeCP2 over-expression. In other words, mis-regulated genes belong to large domains that are relatively rich in mCG and mCAC. These findings fit well with the evidence that MeCP2 may link methylated DNA with the NCoR/SMRT corepressor [13]. Co-repressor recruitment to a genomic domain would be expected to down-regulate genes within that domain according to the density of recruiter binding sites. In the absence of the recruiter, therefore, up-regulation of gene expression would also be proportional to binding site density, as we observe. In addition the results fit with the conclusions of Gabel and colleagues [26] who found repression of long genes by MeCP2. They furthermore accord with a previously published study indicating that transcriptional repression by MeCP2 depends upon domains of DNA methylation, notably in gene bodies [45]. Although we emphasize the correlation with binding site density per unit of DNA length, it is possible that the absolute number of binding sites per gene also contributes to the MeCP2 response. Further work is required to disentangle the roles of these related variables.

The tri-nucleotide mCAC, despite its lower abundance compared with relatively uniformly distributed mCG, correlates strongly with MeCP2 binding and transcriptional mis-regulation in response to altered levels of MeCP2. Superficially, mCG correlates less well than mCAC with changes in gene expression. However, this effect may be exaggerated by the difference in their average densities across the genome. Mis-regulated genes have on average one extra mCAC per 2000 bp when compared with those changing in the opposite direction. Although this is a very small increment, it is robustly measurable compared with the average density of <2 mCAC sequences per kb. In contrast, addition of one mCG per 2000 bp, though an identical change, makes a much smaller difference (<4%) to the already high mCG density in the genome (~15 per kb) and would not therefore be reliably measurable. These considerations leave open the possibility that the small differences in gene expression in response to changing amounts of MeCP2 are equally affected by both mCG and mCAC binding sites.

### Subtle effects on transcription of many genes

While there is no direct evidence that aberrant gene expression is the proximal cause of Rett syndrome or MeCP2 overexpression syndrome, it is noteworthy that thousands of genes, including many implicated in human neuronal disorders, are sensitive to altered levels of MeCP2. Mild mis-regulation on this scale may destabilize neuronal function [26]. It is worth recalling that Rett neurons, though sub-optimal, are viable for many decades. In this sense, the biological defect can be seen as mild, despite the profound effects on higher functions of the brain. The challenge now is to determine how brain function might be affected by a multitude of small discrepancies in gene expression. Overall, the results presented here sustain a coherent view of MeCP2 function: namely that MeCP2 binding at mCG and mCAC sites determines the magnitude of a repressive effect on transcription that is exacerbated by MeCP2 excess and relieved by MeCP2-deficiency. With the benefit of a comprehensive list of MeCP2 target sequences at the molecular level, the predictions of this model can be experimentally tested, clarifying further the role of MeCP2 in regulating transcription in the brain.

## Materials and methods

### Animal care and transgenic mouse lines

All mouse studies were approved and licensed under the UK Animals (Scientific Procedures) Act 1986 and conducted in accordance with guidelines for use and care of laboratory animals. Male *Mecp2*^*STOP/y*^ and corresponding WT littermates [46], male *Mecp2* -/y and WT littermates [38] and *Mecp2* overexpression (OE) mice [40] were used as Western blotting and Real Time PCR controls or for HPLC estimation of RNA/DNA ratio. C57Bl6 male WT 10 week old mice were used for FACS sorting experiments and consecutive WGBS and TAB-seq.

### Protein expression, purification and EMSA

Protein was prepared as described [33]. When examining MeCP2 [1–205] specificity, DNA sequence was varied at the tri-nucleotide indicated in bold (S1 Table). The primary cytosine of this tri-nucleotide was either non-methylated, methylated, or hydroxymethylated. All oligonucleotides were annealed to their complement, ^32^P-labelled and electrophoretic mobility shift assays performed on ice for 30 min using conditions described previously [37]. In competition assays to assess tri-nucleotide-binding preferences of MeCP2 [1–205] a parent 58 bp *Bdnf*-probe, containing the centrally methylated sequence mCGG, was ^32^P-labelled and co-incubated with a 2000-fold excess of cold-competitor DNA bearing one of the sequences described in Fig 1B and 1C. Bound complexes were resolved as described above and levels of competition visualized by Phosphorimager analysis and ImageJ quantification. These experiments were performed in triplicate.

### Transfection ChIP assay

HPLC purified oligonucleotides and corresponding antisense oligonucleotides were purchased from *biomers*.*net*. Some oligonucleotides containing 5hmC were synthesized and characterized as described previously [47]. All oligonucleotide sequences used are listed in S1 Table. Equal amounts of sense and antisense oligonucleotide stocks (100μM) were mixed with 10x Ligation Buffer (*NEB*) in 50μl volumes. Oligonucleotide mix was boiled in a water bath for 8 minutes and cooled to room temperature. Annealed oligonucleotides were cleaned-up with MSB Spin PCRapace cleanup kit (*Invitek*) and diluted to 10μM stocks for transfections. HEK293FT cells (1.5 x 10^6^) were transfected overnight, with 0.5μg of full-length MeCP2 tagged at the N-terminus with EGFP, using Lipofectamine 2000 *(Lifetechnologies)* according to manufacturer’s instructions. After assessment of transfection efficiency (described in [33]), the medium was changed and replaced with annealed unmodified, methylated or hydroxymethylated oligonucleotides [100nM final concentration] using TransIT Oligofect reagent *(Mirus)* for 4 hours. Cells were washed with PBS and harvested by scraping. Cells were then crosslinked with formaldehyde to 1% final concentration for 5 minutes at room temperature and quenched by the addition of glycine to a final concentration of 0.125M for 5 minutes followed by another two washes in PBS. Cell pellets were flash frozen in liquid nitrogen and stored at -80°C or directly used for chromatin isolation and consecutive ChIP with 4μg MeCP2 M6818 antibody *(Sigma)*. Isolated chromatin from transfection ChIP assay was also used for Western blotting to estimate MeCP2 expression levels (S1C Fig). Primer sequences can be found in S2 Table.

### Verification of modified oligonucleotides

Dot blots of modified oligonucleotides and control DNA (Methylated standard kit, *Active Motif*) were generated with Bio-Dot Microfiltration Apparatus (*BioRad*) using manufacturer’s recommendations. Oligonucleotides and control DNA were denaturated by the addition of [0.4M] NaOH, [10mM] EDTA in a total volume of 50μl and boiled for 10 minutes. DNA was neutralized by addition of an equal volume of ice-cold 2M ammonium acetate. Control DNA and oligonucleotides were spotted in duplicate serial dilutions (Control DNA: 50ng, oligonucleotides: 10μM starting concentration). Nitrocellulose membrane was UV auto-crosslinked and then blocked for 30 minutes in 5% non-fat dried milk powder, 0.05% *Tween* 20/ 1x TBS. Primary antibodies were incubated for 45 minutes at room temperature (5hmC: 1:10.000 *Active Motif*; 5mC: 1:500 *Eurogentech*). Secondary *LI-COR* antibodies were incubated in the dark for 30 minutes (donkey anti mouse IRDye 800Cw; donkey anti rabbit IRDye 680; *LI-COR*). Membranes were scanned with a *LI-COR* Odyssey instrument.

### Structural modeling

Modeling was based on the X-ray structure of MeCP2 (PDB code 3C2I) using the program COOT [48]. Atomic coordinates for DNA bases were generated using the ‘mutate’ option. To optimize hydrogen-bonded and van der Waals contacts between protein and different base pair sequences, the conformation of the side chain of R133 was adjusted manually (all other atoms in the structure were left unchanged). The potential role of water molecules in the recognition of different base-pair sequences by MeCP2 was examined by placing a water molecule in the highly conserved and most probable sites of hydration in the major groove of B-DNA as described [49]. All figures were prepared using the graphics program PyMol (*DeLano Scientific*, *San Carlos*, *CA*).

### Mouse brain nuclei isolation and FACS sorting

Brain nuclei isolation and consecutive FACS sorting according to NeuN expression was performed as described previously [16]. 10 week old WT Bl6 male mice were used and 4 brains were pooled for each replicate.

### Whole genome bisulfite and TAB sequencing

Genomic DNA of three replicates of 10 week old male Bl/6 WT dissected hypothalamus samples was prepared with the DNeasy Blood & Tissue Kit *(Qiagen)* and 0.5% unmethylated λ DNA *(Promega)* was spiked in. Equal amounts of genomic DNA were bisulfite converted with the EZ DNA Methylation Gold Kit *(Zymo Research)* and libraries prepared with the TruSeq DNA methylation kit *(Illumina)* according to manufacturer’s instructions. WGBS and TAB sequencing from NeuN positive sorted neuronal nuclei was as described previously [50]. For TAB treatment, half the DNA was glycosylated, TET oxidized and spiked with control DNA. The other half was left untreated and spiked with unmethylated λ DNA (*Promega)*. NGS libraries were prepared with TruSeq DNA Sample Preparation Kit (*Illumina)* according to manufacturer’s instructions. After size selection, all libraries were bisulfite treated with EpiTect Bisulfite Kit (*Qiagen)* and amplified with Pfu Turbo Cx Polymerase *(Stratagene)* for 7 PCR cycles. Cleaned-up libraries were validated on a Bioanalyzer High Sensitivity DNA Chip *(Agilent)* and 100 bp paired-end sequencing performed on an Illumina HiSeq 2000 platform (Wellcome Trust Sanger Institute, Hinxton, UK).

### Normalization of mRNA

Hypothalamus was isolated from 6 week old male WT and *Mecp2* -/y mice in 5 replicates. RNA and DNA were co-isolated with the AllPrep DNA/RNA Mini kit *(Qiagen)* according to manufacturer’s instructions with some modifications. In short, tissue was homogenized in 1ml RLT buffer (spiked with 3 x 10^6^ *Drosophila* S2 cells/10ml RLT buffer) and centrifuged in a *Qiashredder* column *(Qiagen)* for 2 minutes at full speed. The eluted RNA was next subjected to treatment with the DNA-*free* DNA removal kit (*Ambion*) and reverse transcribed with the iScript cDNA synthesis kit (*BioRad*). Real Time quantitative PCR was performed on cDNA and DNA with *Drosophila* and mouse specific mRNA and genomic DNA primers. For analysis, mouse mRNA was normalized to *Drosophila* RNA and analogous mouse DNA was normalized to *Drosophila* DNA. In the final step, corrected mRNA levels were normalized to corrected DNA values. Primer sequences can be found in S3 Table.

### Preparation of total nucleic acid for estimation of RNA versus DNA quantity

Dissected hypothalamus tissue was homogenized in lysis buffer (10mM Tris HCl [pH 7.4], 0.5% SDS, 100mM EDTA, 300μg/ml proteinase K) and incubated at 50°C for 2 hours. Total nucleic acid was recovered from the completely lysed sample by ethanol precipitation in 2 volumes of 100% ethanol at room temperature (for 30 minutes), and pelleting by centrifugation. The pellet was washed once in 2 volumes of 70% ethanol, and the nucleic acid pellet was resuspended in hydrolysis buffer containing 1x DNase I buffer (*NEB*), 1mM zinc sulphate, DNase I (*NEB*) and Nuclease P1 (*Sigma*). After 4 hours, the sample was mixed thoroughly and digested for a further 8 hours. After 12 hours at 37°C, the sample was heated to 92°C for 3 minutes and cooled on ice. Two volumes of 30mM sodium acetate, 1mM zinc sulphate [pH 5.2] were added plus additional Nuclease P1 and the nucleic acids were further digested to deoxyribonucleotide and ribonucleotide 5’ monophosphates for a further 24 hours at 37°C. The samples were then subjected to HPLC as set out below.

### HPLC nucleotide quantifications

UV absorbance was recorded at 276 nm (dCMP, elution time 9.4 minutes), 282 nm (5mdCMP, elution time 17 minutes), 268 nm (dTMP, elution time 21.9 minutes), 260nm AMP and dAMP (elution times 27 minutes and 62.47 minutes) and 254 nm (GMP and dGMP, elution times 11.1 minutes and 29.7 minutes). Extinction coefficients used in nucleotide quantifications were dCMP, 8.86 x 10^3^; 5mdCMP 9.0 x 10^3^; dTMP, dGMP/GMP 12.16 x 10^3^; dAMP/AMP 15.04 x 10^3^. Quantifications were calculated from the area under each peak estimated using Chromeleon software using the respective extinction coefficients.

### Bioinformatics and statistical analyses

#### Genome build and annotations

All data were aligned to the mouse NCBI 37 (mm9) assembly. Gene annotations were obtained from version 67 of the Ensembl database.

#### ChIP-seq

MeCP2 Chip and Input datasets were downloaded from the Gene Expression Omnibus (GEO) under the accession numbers GSE66868 [27] and GSE60062 [26]. Raw sequencing reads were first trimmed and filtered using Trimmomatic v0.32 [51] then aligned with bwa using the samse algorithm. Alignments were then filtered to remove reads classed as duplicates, non-unique or those that fell in the blacklisted regions outlined by the Encode project [52]. Alignments were converted to bigWig files using the deepTools package for genome wide visualization and analysis. Read counts were normalized to RPKM to account for differences in library size. The bigwigCompare tool was used to calculate a log2 ratio of MeCP2 ChIP/Input signal across the genome.

#### Whole genome bisulfite sequencing of mouse hypothalamus

Quality trimming, filtering and adapter removal were performed by Trimmomatic v0.32 prior to alignment. The software package Bismark v0.15 was used to map reads to the mm9 genome, remove duplicate alignments and extract methylation calls [53].

#### BS-seq and TAB-seq

Processed bisulfite and TAB-seq datasets containing aligned methylation calls were obtained from the GEO records GSM1173786_allC.MethylC-Seq_mm_fc_male_7wk_neun_pos and GSM1173795_allC.TAB-Seq_mm_fc_6wk respectively. Percentage (%) DNA methylation at a given site *i* corresponds to the ratio of mC basecalls for that site to the count of all reads mapping to that site (*m*^*i*^ = *mC* / *C*). Context-specific % mean methylation for a given region (i.e. bin or gene) was defined as ${\overset{´}{m}}_{CX}=\frac{\sum_{i}m_{CX}^{i}}{N_{CX}}$, where *N*_*CX*_ is the number of C’s within the region, occurring in context *CX*. In addition, context-specific methylation density was defined as $\frac{N_{CX}\cdot {\overset{´}{m}}_{CX}}{L}$, where L is the length of the region.

#### RNA-seq

Raw reads from mouse hypothalamus were downloaded from the GEO under the accession no. GSE66870 [27]. Trimmomatic v0.32 was used to remove adaptor contamination and to trim low quality reads. Reads were mapped to the genome using STAR v 2.4.2a [54]. Alignments were then filtered to remove non-unique and blacklisted reads. HTseq-count v0.6.0 was used to quantify read counts over gene exons in the union mode.

#### Correlation between MeCP2 ChIP-Seq and Input read counts (Fig 3E and 3F)

For the MeCP2 ChIP and Input sample of the Chen dataset [27] we computed the number of reads that mapped to 10 kb windows covering the entire genome. For this we have shifted the reads by 134 bp which corresponds to the estimated fragment length determined by MACS. A Kernel density estimate was applied.

#### Correlation between log2(MeCP2 ChIP/Input) and DNA methylation (Fig 4E and 4F and S3D–S3L Fig)

We calculated read coverage values and context-dependent methylation densities for 1 kb windows across the genome. Regions were ordered by their log2(MeCP2 ChIP/Input) signal and mean methylation densities were calculated for groups of 1000 regions. Datasets used are [27] and hypothalamus WGBS (this study) or cortex [3,26].

#### MeCP2 summit analysis (Fig 3G and 3H and S2B and S2C Fig)

Summits of MeCP2 ChIP enrichment over Input were defined using MACS and the method described in [26] for both MeCP2 datasets ([26,27]). We used the Bioconductor package *seqplots* v*1*.*4*.*0* to plot methylation density across the summits for various cytosine contexts. Mean methylation densities were calculated for 20 bp windows across the summits extended by 4 kb on either side.

#### Rolling mean plots (Fig 4A–4D, S3A–S3C Fig)

Genes were sorted according to their MeCP2/Input enrichment and rolling means of methylation density were applied over subsets of 400 genes with a step of 80 genes. Datasets used are [27] and hypothalamus WGBS (this study).

The ChIP-seq dataset from cortex was combined with bisulfite analysis from [3] (Fig 3G, S2C, S3A–S3C, S3F and S3H Figs) and the hypothalamus ChIP-seq dataset [27] was correlated with bisulfite data from hypothalamus WGBS (this study) (Figs 3H and 4A–4C and S2B, S3D and S3I–S3L Figs) or bisulfite data from [3] (S3E and S3G Fig).

#### MSR (Fig 4G and 4H and S3M–S3O Fig)

We used the MSR tool to find domains of MeCP2 enrichment and depletion relative to the Input sample [36]. As background, we used a mappability map for the m9 genome (parameters: L = 45, P-value threshold: 1e-6). For each significantly enriched or depleted segment we determined the methylation densities and averaged over segments with same scale and enrichment score. Datasets used are [27] and hypothalamus WGBS (this study).

#### Differential expression analysis (Fig 5 and S5 Fig)

We used DeSeq2 v1.8.1 [55] to determine mis-regulated genes in KO and OE. To account for the observed 15% reduction of total mRNA in the KO samples, we first used the DESeq function estimateSizeFactors to normalize the data sets and subsequently multiplied the obtained size factors for the KO samples by 1.15. An adjusted p-value threshold of <0.05 was used to determine up- or down-regulated genes. For an exemplary group of genes for which little change is observed between conditions we used p_adj >0.5 with a log2FoldChange <0.01. We further filtered out protein-coding genes with constitutively low expression (TPM <5 in all samples) and genes with the lowest Input coverage. We retained 12,510 protein-coding genes, of which 403 showed higher, and 8363 lower expression in KO vs WT, respectively. In addition, we found 1372 to have higher and 2719 to have lower expression in OE relative to WT. Dataset used is [27].

#### Aggregated signal plots across gene features (Fig 5A–5F)

We used *seqplots* to generate aggregate plots at transcription start and end sites of protein coding genes and annotated CGIs. Mean values for log2(MeCP2 ChIP/Input) and methylation densities were calculated over 1 kb windows for each set of genes and 100 bp windows for CGIs. Datasets used are [27] and hypothalamus WGBS (this study).

## Supporting information

S1 Fig *In vivo* assay to assess binding specificities of MeCP2. (A) Transfection efficiency of HEK293FT cells with MeCP2-GFP (left panel) and 5’ Cy3 labeled oligonucleotides (right panel). Bright field images of cells after transfections were taken and merged with the corresponding fluorescence channels for GFP and Cy3. All images were taken at a 20x magnification. (B) Dot blot of fully methylated (5mC) and hydroxymethylated (5hmC) control DNA (upper panel) and synthesized oligonucleotides used in the *in vivo* transfection assays in three dilutions (lower panel, upper part: mCG/mCA; lower panel, lower part: hmCG and hmCA). The membrane was probed with antibodies against 5mC (red fluorescence) and 5hmC (green fluorescence). (C) Western blot of chromatin from HEK293FT cells transfected with MeCP2-GFP (+) and various modified oligonucleotides to control for equal MeCP2-GFP transfection efficiency. (-) cells lacking transfected MeCP2-GFP demonstrate that endogenous MeCP2 does not significantly contribute to ChIP of transfected mCG oligonucleotide in the *in vivo* assay (See Fig 2E, grey bar). UN: unmethylated. The membrane was probed with antibodies against the N-terminus of MeCP2 (green fluorescence) and C terminal histone H3 (red fluorescence) as a loading control. The asterisk indicates the expected position of endogenous MeCP2.
(TIF)

S2 Fig MeCP2 binding specificities in the brain. (A) Depletion of Input corrected MeCP2 ChIP-seq signal over all annotated CpG islands (CGI) (datasets from [26,27]). (B) Enrichment of mCG and mCA di-nucleotides at summits of MeCP2 binding measured by ChIP-seq. Datasets [27] and hypothalamus WGBS from this study were used for the analysis. (C) No enrichment of mCAX triplets in summit analysis of random regions measured by ChIP-seq (datasets [3,26]).
(TIF)

S3 Fig MeCP2 binding correlates with mCG and mCAC and binds in large domains extending the gene scale. (A) Density of mCG/mCAC or unmethylated CG per 1kb as a function of ChIP-seq Input coverage in protein-coding genes. The density of tick marks on the x-axis represents the distribution of genes with respect to Input read coverage (datasets from [3,26]). (B) As in (A) but for MeCP2 ChIP-seq read coverage on the x-axis. (C) As in (B) but with log2(MeCP2 ChIP/Input) shown on the x-axis (datasets from [3,26]). (D) Relationship between mCGX density/kb and MeCP2 occupancy corrected for Input in genome-wide 1 kb windows. Datasets from [27] and WGBS hypothalamus from this study were used for the analysis. (E-F) Relationship between mCAX density/kb and MeCP2 occupancy corrected for Input in genome-wide 1 kb windows (datasets from [3,26,27]). (G-H) Relationship between mCG, hmCG and mCAC density/kb and MeCP2 occupancy corrected for Input in genome-wide 1 kb windows (datasets from [3,26,27]). (I-L) Relationship between unmethylated CAX (I), CCX (J), CGX (K) and CTX (L) density/kb and MeCP2 occupancy corrected for Input in genome-wide 1 kb windows. Datasets from [27] and WGBS hypothalamus from this study were used for the analysis. (M-O) Domains of MeCP2 enrichment and depletion as determined by multiscale representation (MSR). Heatmaps showing the mean methylation density of segments binned by their scale (x-axis) and enrichment scores (y-axis). Positive scores on the y-axis indicate enrichment, negative scores MeCP2 depletion. Plots are colored by mCG density (M), mCAC density (N) and median GC content in % (O). Datasets from [27] and hypothalamus WGBS from this study were used for the analysis. (P-Q) Box plots for AT-rich sequence runs flanking mCG (P) or mCAC (Q) regions of MeCP2 enrichment in frontal cortex (datasets [3,26]).
(TIF)

S4 Fig MeCP2 occupancy correlates with the direction and magnitude of transcriptional de-regulation caused by MeCP2 excess or deficiency. (A) HPLC analysis to establish total RNA/DNA ratios in hypothalamus triplicates of *Mecp2* WT, KO and OE adult male mice. Ratios for Guanosine (RNA) to Deoxycytidine (DNA) and Adenosine (RNA) to Deoxyguanosine (DNA) nucleosides are shown. (B) Schematic showing the experimental setup for mRNA normalization using *Drosophila* spike-in to control for technical error and normalization to brain cell number by paralleled DNA isolation. Quantitative Real Time PCR was performed with primers against *Drosophila* and mouse genomic DNA and *Drosophila* and mouse mRNA. All primer sequences can be found in S3 Table. (C-F) Quantitative Real Time PCR of up-regulated and down-regulated genes in 5 replicates of WT and *Mecp2* KO adult hypothalamus as identified by the original RNA-seq normalization in [26]. By normalizing to brain cell number and correcting for technical loss in RNA and DNA isolation procedures as described in (B), these genes still remain up- (C-D) and down-regulated (E-F), respectively. (G-I) Quantitative Real Time PCR of housekeeping genes in 5 replicates of WT and *Mecp2* KO adult hypothalamus using the experimental setup discussed in (B). Mouse mRNA primers for *Cyclophilin A* (G), *Hprt* (H) and *Gapdh* (I) were used. All error bars represent ± SD. Students unpaired t-test: ns p>0.05; * p<0.05; ** p<0.01; *** p<0.001. All primer sequences can be found in S3 Table.
(TIF)

S5 Fig MeCP2 repressed genes are enriched in large genomic domains occupied by MeCP2 and correspond to neuronal GO-terms. (A) Pie chart showing up-regulated (purple), unchanged (grey) and down-regulated (green) genes in *Mecp2* KO adult hypothalamus as a percentage of all genes found in MeCP2 depleted regions. (B) Pie chart showing up-regulated (purple), unchanged (grey) and down-regulated (green) genes in *Mecp2* KO adult hypothalamus as a percentage of all genes found in MeCP2 enriched regions. (C) Scatterplot showing log2 fold expression changes and log2(MeCP2 ChIP/Input) taking into account a 15% down-regulation of total RNA in *Mecp2* KO adult hypothalamus. (D) Scatterplot showing expression changes and log2(MeCP2 ChIP/Input) in *Mecp2* OE adult hypothalamus. Datasets from [27] were used for the analysis. (E) Scatterplot showing reciprocity of direction of gene de-regulation in a MeCP2 dependent manner. The genes going up in *Mecp2* KO/WT (purple) show most down-regulation in OE/WT and *vice versa*, respectively. (F-G) Scatterplot and histogram of de-regulated genes plotted according to their mCG (F) and mCAC (G) densities. All de-regulated and unchanged genes exhibit comparable levels of mCG density (F), but the genes up-regulated in *Mecp2* KO show an enrichment in mCAC density (G). (H) Box plot showing gene length in genes up, unchanged and down in KO. (I) GO-term analysis of up-regulated genes in KO.
(TIF)

S6 Fig Hypothetical Modeling of MeCP2 with methylated CAX and hydroxymethylated CAC. (A) Model of MeCP2 [77–167] interaction with double strand DNA containing mCAT tri-nucleotide. R111, R133 and D121 amino acids are shown in pink. Green sphere: methyl group; grey sphere: water molecule; yellow sphere: artificially placed water molecule; green dotted lines: favorable interactions, black dotted lines: newly modeled interactions, grey dotted lines: hydrogen bonds. (B) As (A) but in a mCAA context; Y123 is shown in pink. Red dotted lines: unfavorable interactions. (C) As (A, B) but in a mCAG context. (D) Model of MeCP2 [77–167] binding to hmCG. Red sphere: hydroxymethyl group. (E) As (D) but in hmCAC context. Rationale for hypothetical modelling: R133 makes essential hydrogen bonds with one guanine base in the mCG complex and also provides salt bridges with a cytosine methyl group (Fig 6A) [42]. The equivalent guanine residue on the other DNA strand of the mCG dyad is also hydrogen bonded to an arginine: residue R111. Mutations in either R133 or R111 cause Rett syndrome, but despite their related roles, the conformations of R111 and R133 are very different. Whereas the R111 side-chain adopts an extended all-trans conformation that is “pinned” by hydrogen bonds with D121, R133 is relatively unconstrained by surrounding amino acids (Fig 6A). We therefore asked if R133 could potentially interact with mCAC in the existing X-ray structure [42]. Indeed, by extending the side-chain and tilting the guanidinium group, R133 can make permitted stabilizing hydrogen bonds with the guanine base that is paired with the third cytosine in the mCAC tri-nucleotide (Fig 6B). An equivalent interaction is also possible with the complementary adenine in mCAT tri-nucleotide (S6A Fig). Importantly, interactions with pyrimidine bases are sterically unfavorable, arguing that mCAA or mCAG are unlikely to interact with the MBD, as is observed experimentally (S6B-C Fig). Modeling of hmCG binding indicates that the presence of the cytosine hydroxyl group would not be accommodated due to the close proximity of this polar group with the guanidinium group of R111 (S6D Fig). Binding of hmCAC is allowed, however, due to tilting of the R133 side-chain and the formation of hydrogen bonds with guanine on the opposite DNA strand (S6E Fig).
(TIF)

S1 Table Oligonucleotide sequences for EMSA and transfection assay. Oligonucleotide sequences for EMSA *Bdnf* probe (58bp) and transfection assays for three CAC modifications (109bp), *Bdnf* single modification in a CG context (104bp) and *Bdnf* single modification in a CAX context (105bp) are shown.
(DOCX)

S2 Table Primer sequences for ChIP. T3 and M13-20 standard primers used for ChIP PCR in transfection assays are shown.
(DOCX)

S3 Table Primer sequences for mRNA normalization. Primer sequences for *Mus musculus* (*Mm*) and *Drosophila melanogaster* (*Dm*) mRNA normalization experiments are shown.
(DOCX)
