# Supplementary figures and images for: MeCP2 recognizes cytosine methylated tri-nucleotide and di-nucleotide sequences to tune transcription in the mammalian brain
Source: PLoS Genet. 2017 May 12;13(5):e1006793. doi: 10.1371/journal.pgen.1006793 (PMC5446194; doi:10.1371/journal.pgen.1006793)

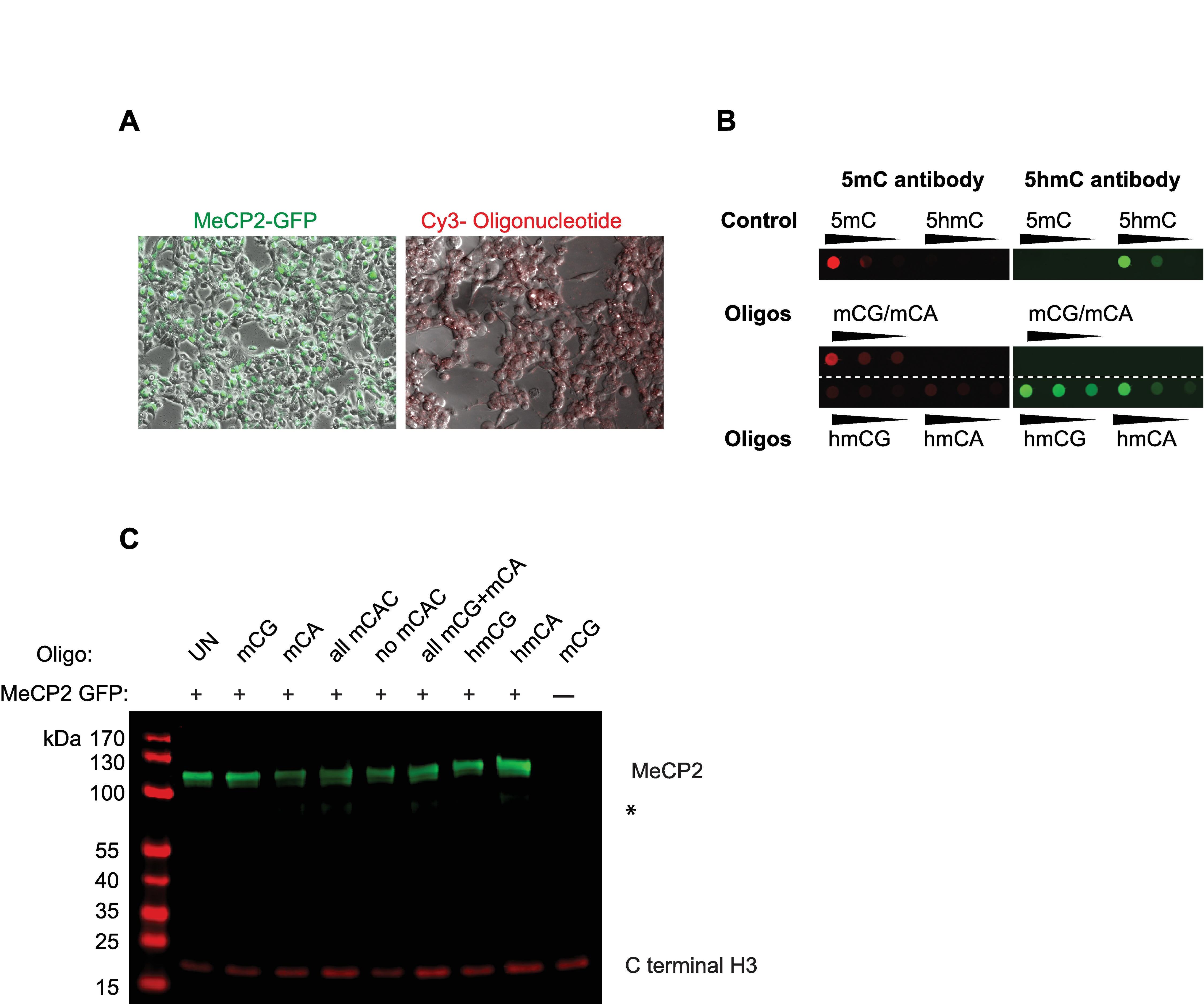

Supplement: S1 Fig — (A) Transfection efficiency of HEK293FT cells with MeCP2-GFP (left panel) and 5’ Cy3 labeled oligonucleotides (right panel). Bright field images of cells after transfections were taken and merged with the corresponding fluorescence channels for GFP and Cy3. All images were taken at a 20x magnification. (B) Dot blot of fully methylated (5mC) and hydroxymethylated (5hmC) control DNA (upper panel) and synthesized oligonucleotides used in the in vivo transfection assays in three dilutions (lower panel, upper part: mCG/mCA; lower panel, lower part: hmCG and hmCA). The membrane was probed with antibodies against 5mC (red fluorescence) and 5hmC (green fluorescence). (C) Western blot of chromatin from HEK293FT cells transfected with MeCP2-GFP (+) and various modified oligonucleotides to control for equal MeCP2-GFP transfection efficiency. (-) cells lacking transfected MeCP2-GFP demonstrate that endogenous MeCP2 does not significantly contribute to ChIP of transfected mCG oligonucleotide in the in vivo assay (See Fig 2E, grey bar). UN: unmethylated. The membrane was probed with antibodies against the N-terminus of MeCP2 (green fluorescence) and C terminal histone H3 (red fluorescence) as a loading control. The asterisk indicates the expected position of endogenous MeCP2. (TIF) [file pgen.1006793.s001.tif]

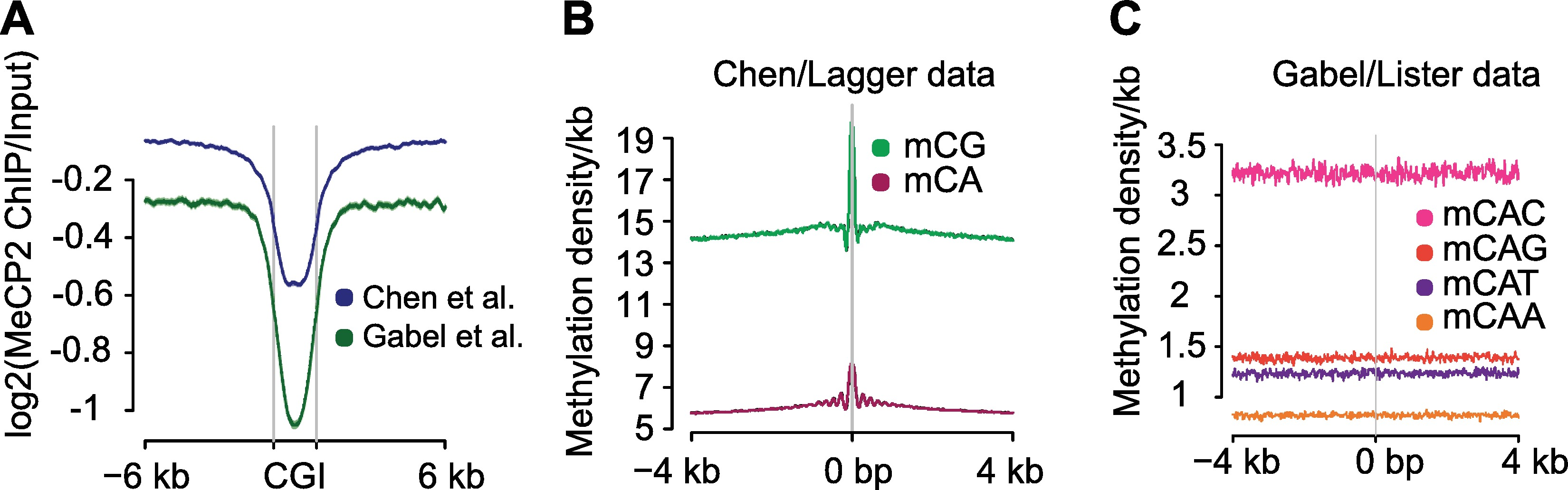

Supplement: S2 Fig — (A) Depletion of Input corrected MeCP2 ChIP-seq signal over all annotated CpG islands (CGI) (datasets from [26,27]). (B) Enrichment of mCG and mCA di-nucleotides at summits of MeCP2 binding measured by ChIP-seq. Datasets [27] and hypothalamus WGBS from this study were used for the analysis. (C) No enrichment of mCAX triplets in summit analysis of random regions measured by ChIP-seq (datasets [3,26]). (TIF) [file pgen.1006793.s002.tif]

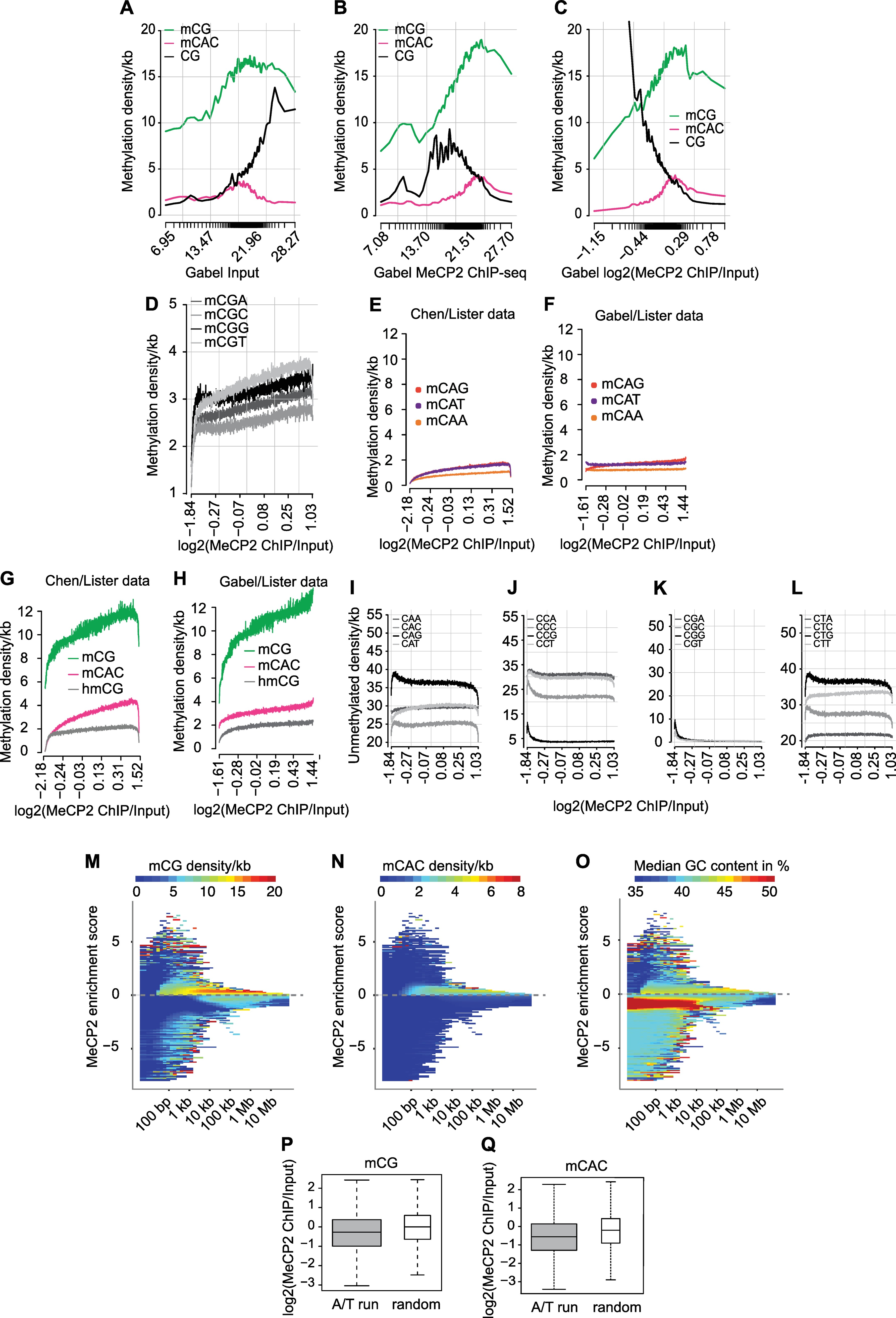

Supplement: S3 Fig — (A) Density of mCG/mCAC or unmethylated CG per 1kb as a function of ChIP-seq Input coverage in protein-coding genes. The density of tick marks on the x-axis represents the distribution of genes with respect to Input read coverage (datasets from [3,26]). (B) As in (A) but for MeCP2 ChIP-seq read coverage on the x-axis. (C) As in (B) but with log2(MeCP2 ChIP/Input) shown on the x-axis (datasets from [3,26]). (D) Relationship between mCGX density/kb and MeCP2 occupancy corrected for Input in genome-wide 1 kb windows. Datasets from [27] and WGBS hypothalamus from this study were used for the analysis. (E-F) Relationship between mCAX density/kb and MeCP2 occupancy corrected for Input in genome-wide 1 kb windows (datasets from [3,26,27]). (G-H) Relationship between mCG, hmCG and mCAC density/kb and MeCP2 occupancy corrected for Input in genome-wide 1 kb windows (datasets from [3,26,27]). (I-L) Relationship between unmethylated CAX (I), CCX (J), CGX (K) and CTX (L) density/kb and MeCP2 occupancy corrected for Input in genome-wide 1 kb windows. Datasets from [27] and WGBS hypothalamus from this study were used for the analysis. (M-O) Domains of MeCP2 enrichment and depletion as determined by multiscale representation (MSR). Heatmaps showing the mean methylation density of segments binned by their scale (x-axis) and enrichment scores (y-axis). Positive scores on the y-axis indicate enrichment, negative scores MeCP2 depletion. Plots are colored by mCG density (M), mCAC density (N) and median GC content in % (O). Datasets from [27] and hypothalamus WGBS from this study were used for the analysis. (P-Q) Box plots for AT-rich sequence runs flanking mCG (P) or mCAC (Q) regions of MeCP2 enrichment in frontal cortex (datasets [3,26]). (TIF) [file pgen.1006793.s003.tif]

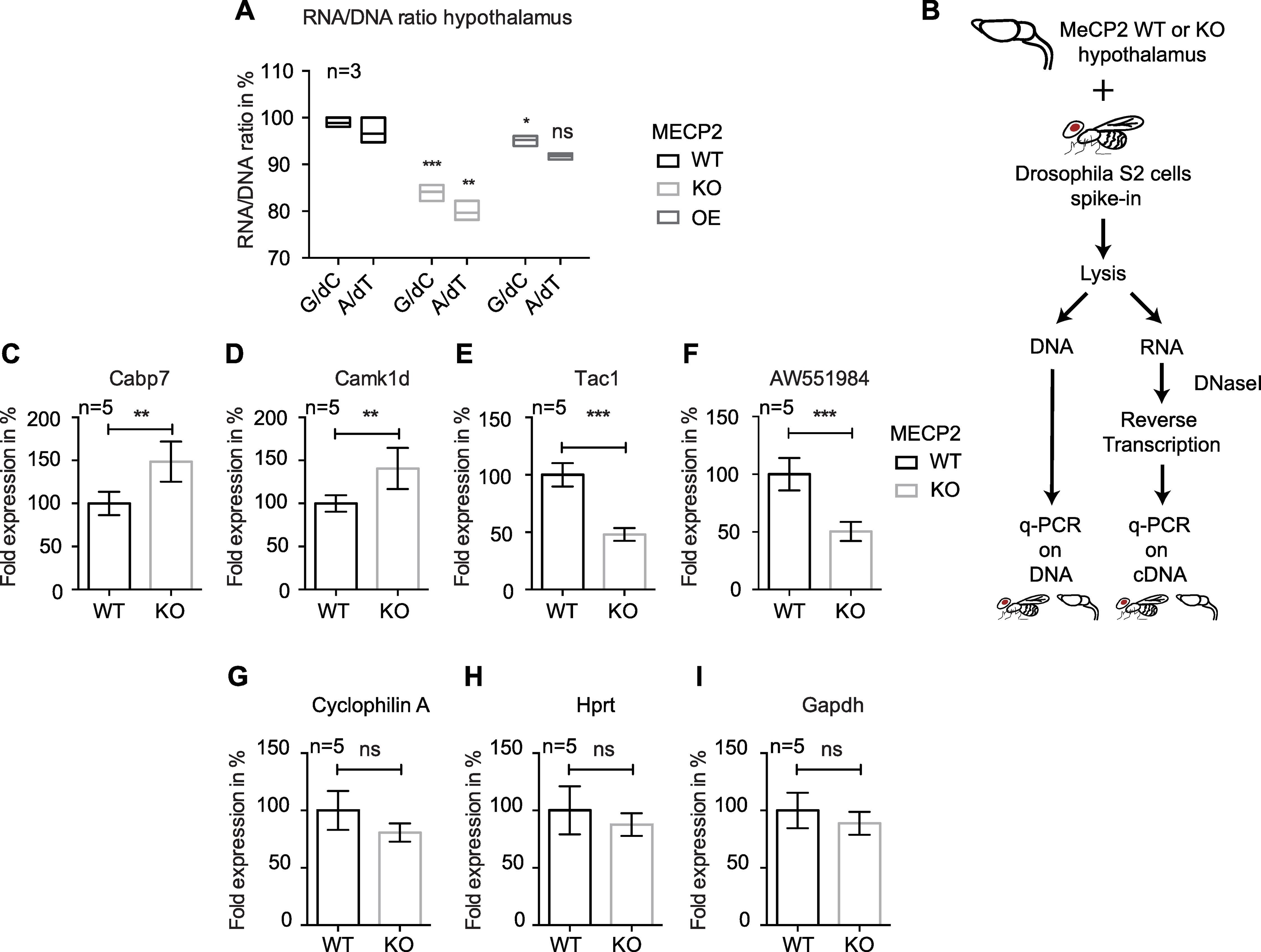

Supplement: S4 Fig — (A) HPLC analysis to establish total RNA/DNA ratios in hypothalamus triplicates of Mecp2 WT, KO and OE adult male mice. Ratios for Guanosine (RNA) to Deoxycytidine (DNA) and Adenosine (RNA) to Deoxyguanosine (DNA) nucleosides are shown. (B) Schematic showing the experimental setup for mRNA normalization using Drosophila spike-in to control for technical error and normalization to brain cell number by paralleled DNA isolation. Quantitative Real Time PCR was performed with primers against Drosophila and mouse genomic DNA and Drosophila and mouse mRNA. All primer sequences can be found in S3 Table. (C-F) Quantitative Real Time PCR of up-regulated and down-regulated genes in 5 replicates of WT and Mecp2 KO adult hypothalamus as identified by the original RNA-seq normalization in [26]. By normalizing to brain cell number and correcting for technical loss in RNA and DNA isolation procedures as described in (B), these genes still remain up- (C-D) and down-regulated (E-F), respectively. (G-I) Quantitative Real Time PCR of housekeeping genes in 5 replicates of WT and Mecp2 KO adult hypothalamus using the experimental setup discussed in (B). Mouse mRNA primers for Cyclophilin A (G), Hprt (H) and Gapdh (I) were used. All error bars represent ± SD. Students unpaired t-test: ns p>0.05; * p<0.05; ** p<0.01; *** p<0.001. All primer sequences can be found in S3 Table. (TIF) [file pgen.1006793.s004.tif]

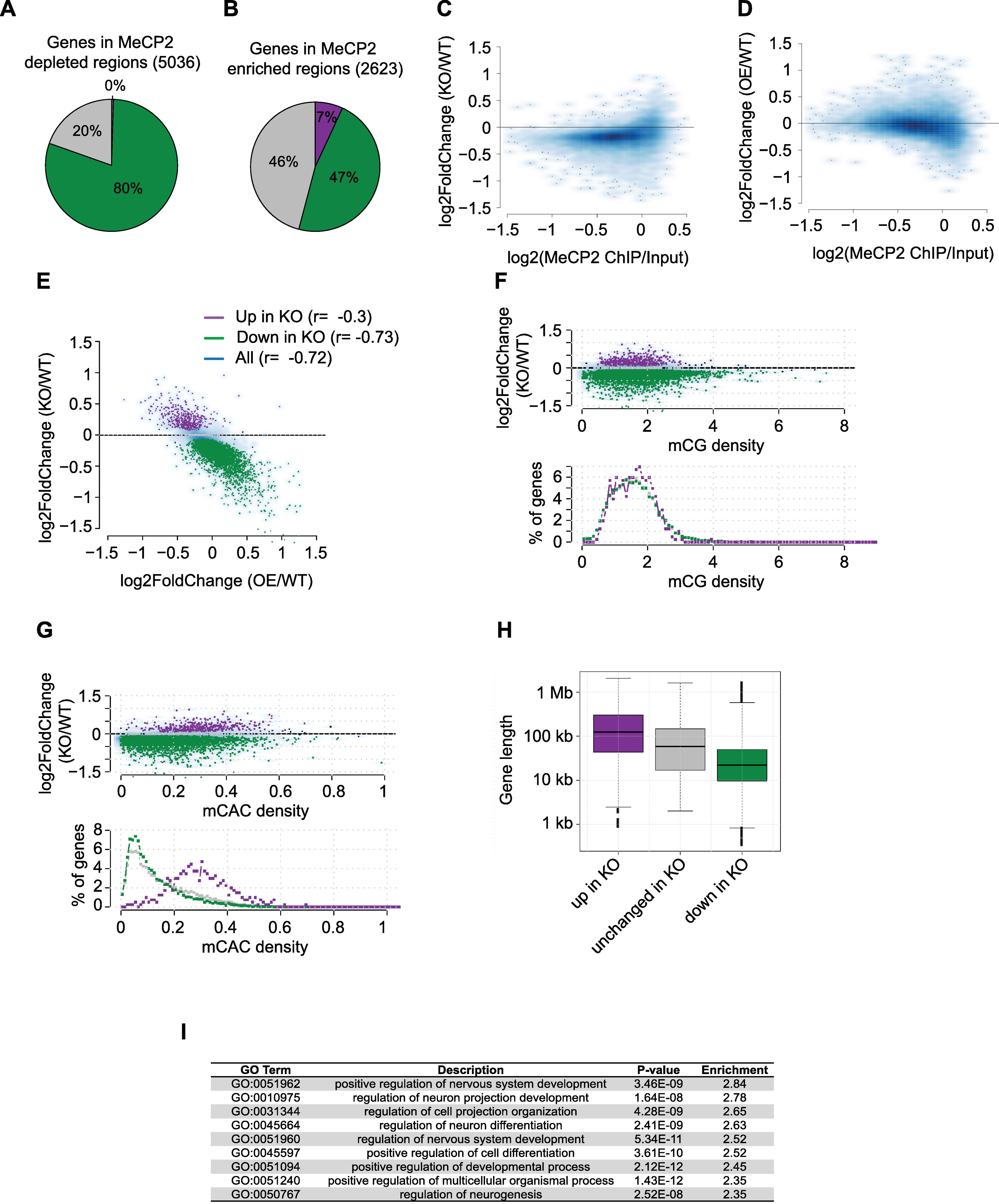

Supplement: S5 Fig — (A) Pie chart showing up-regulated (purple), unchanged (grey) and down-regulated (green) genes in Mecp2 KO adult hypothalamus as a percentage of all genes found in MeCP2 depleted regions. (B) Pie chart showing up-regulated (purple), unchanged (grey) and down-regulated (green) genes in Mecp2 KO adult hypothalamus as a percentage of all genes found in MeCP2 enriched regions. (C) Scatterplot showing log2 fold expression changes and log2(MeCP2 ChIP/Input) taking into account a 15% down-regulation of total RNA in Mecp2 KO adult hypothalamus. (D) Scatterplot showing expression changes and log2(MeCP2 ChIP/Input) in Mecp2 OE adult hypothalamus. Datasets from [27] were used for the analysis. (E) Scatterplot showing reciprocity of direction of gene de-regulation in a MeCP2 dependent manner. The genes going up in Mecp2 KO/WT (purple) show most down-regulation in OE/WT and vice versa, respectively. (F-G) Scatterplot and histogram of de-regulated genes plotted according to their mCG (F) and mCAC (G) densities. All de-regulated and unchanged genes exhibit comparable levels of mCG density (F), but the genes up-regulated in Mecp2 KO show an enrichment in mCAC density (G). (H) Box plot showing gene length in genes up, unchanged and down in KO. (I) GO-term analysis of up-regulated genes in KO. (TIF) [file pgen.1006793.s005.tif]

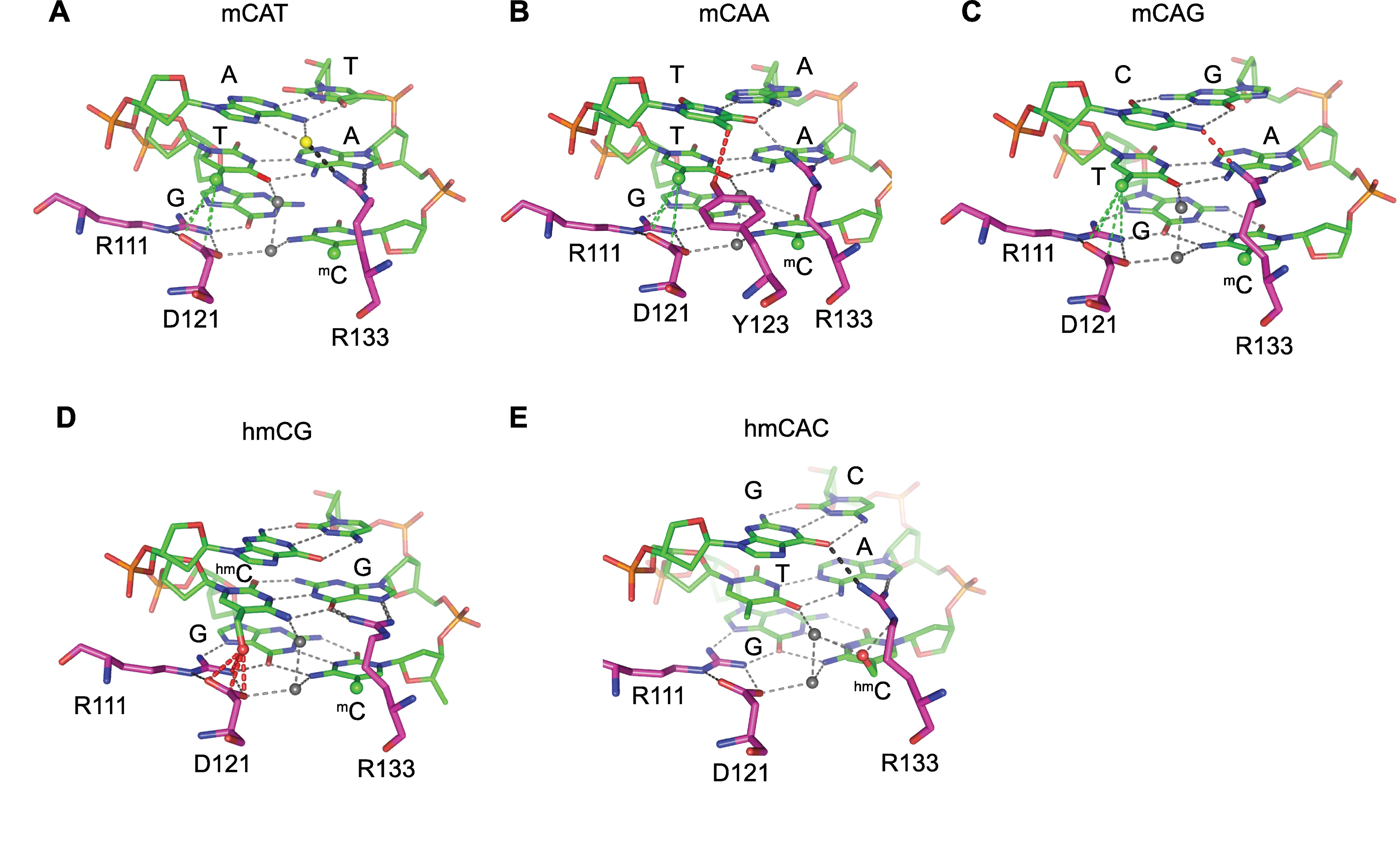

Supplement: S6 Fig — (A) Model of MeCP2 [77–167] interaction with double strand DNA containing mCAT tri-nucleotide. R111, R133 and D121 amino acids are shown in pink. Green sphere: methyl group; grey sphere: water molecule; yellow sphere: artificially placed water molecule; green dotted lines: favorable interactions, black dotted lines: newly modeled interactions, grey dotted lines: hydrogen bonds. (B) As (A) but in a mCAA context; Y123 is shown in pink. Red dotted lines: unfavorable interactions. (C) As (A, B) but in a mCAG context. (D) Model of MeCP2 [77–167] binding to hmCG. Red sphere: hydroxymethyl group. (E) As (D) but in hmCAC context. Rationale for hypothetical modelling: R133 makes essential hydrogen bonds with one guanine base in the mCG complex and also provides salt bridges with a cytosine methyl group (Fig 6A) [42]. The equivalent guanine residue on the other DNA strand of the mCG dyad is also hydrogen bonded to an arginine: residue R111. Mutations in either R133 or R111 cause Rett syndrome, but despite their related roles, the conformations of R111 and R133 are very different. Whereas the R111 side-chain adopts an extended all-trans conformation that is “pinned” by hydrogen bonds with D121, R133 is relatively unconstrained by surrounding amino acids (Fig 6A). We therefore asked if R133 could potentially interact with mCAC in the existing X-ray structure [42]. Indeed, by extending the side-chain and tilting the guanidinium group, R133 can make permitted stabilizing hydrogen bonds with the guanine base that is paired with the third cytosine in the mCAC tri-nucleotide (Fig 6B). An equivalent interaction is also possible with the complementary adenine in mCAT tri-nucleotide (S6A Fig). Importantly, interactions with pyrimidine bases are sterically unfavorable, arguing that mCAA or mCAG are unlikely to interact with the MBD, as is observed experimentally (S6B-C Fig). Modeling of hmCG binding indicates that the presence of the cytosine hydroxyl group would not be accommodated due [file pgen.1006793.s006.tif]
