# Supplementary material for: MeCP2 recognizes cytosine methylated tri-nucleotide and di-nucleotide sequences to tune transcription in the mammalian brain
Source: PLoS Genet. 2017 May 12;13(5):e1006793. doi: 10.1371/journal.pgen.1006793 (PMC5446194; doi:10.1371/journal.pgen.1006793)
Supplement: S1 Table — Oligonucleotide sequences for EMSA Bdnf probe (58bp) and transfection assays for three CAC modifications (109bp), Bdnf single modification in a CG context (104bp) and Bdnf single modification in a CAX context (105bp) are shown. (DOCX) [file pgen.1006793.s007.docx]

S1 Table Oligonucleotide sequences for EMSA and transfection assay.

| EMSA *Bdnf* probe (58 bp): |
| --- |
| 5’-AAGCATGCAATGCCCTGGAA**CGG**AATTCTTCTAATAAAAGATGTATCATT  TTAAATGC-3’ |
| Transfection Assay CAC modifications (109 bp): |
| 5’-ATGCTAATTAACCCTCACTAAAGGGAACTCGAGA**CAC**CGGAGAATTCAC  AT**CAC**CGGTGAATCAGTGCTACCCG**CAC**GTGCACTGGATCCACTGGCCG  TCGTTTTACAA-3’ |
| Transfection Assay *Bdnf* single modification CG context (104 bp): |
| 5’-ATGCTAATTAACCCTCACTAAAGGGAAAAGCATGCAATGCCCTGGAA**CG**  GAATTCTTCTATAAAAGATGTATCATTTTAAATGCACTGGCCGTCGTTTTAC  AA-3’ |
| Transfection Assay *Bdnf* single modification CAX context (105 bp): |
| 5’-ATGCTAATTAACCCTCACTAAAGGGAAAAGCATGCAATGCCCTGGAA  **CAX**GAATTCTTCTAATAAAAGATGTATCATTTTAAATGCACTGGCCGTCG  TTTTACAA-3’ |
