# Supplementary material for: MeCP2 recognizes cytosine methylated tri-nucleotide and di-nucleotide sequences to tune transcription in the mammalian brain
Source: PLoS Genet. 2017 May 12;13(5):e1006793. doi: 10.1371/journal.pgen.1006793 (PMC5446194; doi:10.1371/journal.pgen.1006793)
Supplement: S2 Table — T3 and M13-20 standard primers used for ChIP PCR in transfection assays are shown. (DOCX) [file pgen.1006793.s008.docx]

S2 Table Primer sequences for ChIP.

| T3 | 5’-AATTAACCCTCACTAAAGGGAA-3’ |
| --- | --- |
| M13-20 | 5’-GTAAAACGACGGCCAGT-3’ |
