# Supplementary material for: MeCP2 recognizes cytosine methylated tri-nucleotide and di-nucleotide sequences to tune transcription in the mammalian brain
Source: PLoS Genet. 2017 May 12;13(5):e1006793. doi: 10.1371/journal.pgen.1006793 (PMC5446194; doi:10.1371/journal.pgen.1006793)
Supplement: S3 Table — Primer sequences for Mus musculus (Mm) and Drosophila melanogaster (Dm) mRNA normalization experiments are shown. (DOCX) [file pgen.1006793.s009.docx]

S3 Table Primer sequences for mRNA normalization.

| *Mm* mRNA Hprt F | 5’ GCTGGTGAAAAGGACCTCT 3’ |
| --- | --- |
| *Mm* mRNA Hprt R | 5’ CACAGGACTAGAACACCTGC 3’ |
| *Mm* mRNA Cyclophilin A F | 5’ TCGAGCTCTGAGCACTGGAG 3’ |
| *Mm* mRNA Cyclophilin A R | 5’ CATTATGGCGTGTAAAGTCACCA 3’ |
| *Mm* mRNA Gapdh F | 5’ GTCGTGTGAACGGATTTG 3’ |
| *Mm* mRNA Gapdh R | 5’ GACTCCACGACATACTCA 3’ |
| \| *Mm* mRNA Cabp7 F \| \| --- \| | 5’ TGATGTACCGGGGCATCTAC 3’ |
| *Mm* mRNA Cabp7 R | 5’ TTGAAGGCCTCTCGGATCTC 3’ |
| *Mm* mRNA Camk1d F | 5’ TGCCCAGATCCGGAAGAATT 3’ |
| *Mm* mRNA Camk1d R | 5’ GGTTGCTTGAGACACTTGCA 3’ |
| *Mm* mRNA AW551984 F | 5’ AATGCAGATGGTTCCTGGGA 3’ |
| *Mm* mRNA AW551984 R | 5’ CCAGGCTGAGGGTTCTAACA 3’ |
| *Mm* mRNA Tac1 F | 5’ GGACATGGCCAGATCTCTCA 3’ |
| *Mm* mRNA Tac1 R | 5’ TTTCGTAGTTCTGCATCGCG 3’ |
| *Mm* DNA Bdnf F | 5’-TTCGATTCACGCAGTTGTTC-3’ |
| *Mm* DNA Bdnf R | 5’-CTGAGCCAGTTACGTGACCA-3’ |
| *D. mel* mRNA CG11076 F | 5’ TGTCCCAAGGGTGCAGATCC 3’ |
| *D. mel* mRNA CG11076 R | 5’ GGAAGCCTAATGCGCTTGCC 3’ |
| *D. mel* DNA F | 5’ CACGGATTCATTCGTAGAGC 3’ |
| *D. mel* DNA R | 5’ GACGAAAGCATATCAGCCAG 3’ |
